# Patients and animal models of CNG**β**1-deficient retinitis pigmentosa support gene augmentation approach

**DOI:** 10.1172/JCI95161

**Published:** 2017-11-20

**Authors:** Simon M. Petersen-Jones, Laurence M. Occelli, Paige A. Winkler, Winston Lee, Janet R. Sparrow, Mai Tsukikawa, Sanford L. Boye, Vince Chiodo, Jenina E. Capasso, Elvir Becirovic, Christian Schön, Mathias W. Seeliger, Alex V. Levin, Stylianos Michalakis, William W. Hauswirth, Stephen H. Tsang

**Affiliations:** 1 Department of Small Animal Clinical Sciences, Michigan State University, East Lansing, Michigan, USA.; 2 Department of Ophthalmology Pathology & Cell Biology, College of Physicians and Surgeons, Columbia University, New York, New York, USA.; 3 Thomas Jefferson University, Philadelphia, Pennsylvania, USA.; 4 Department of Ophthalmology, University of Florida, Gainesville, Florida, USA.; 5 Ocular Genetics, Wills Eye Hospital (WEH), Philadelphia, Pennsylvania, USA.; 6 Center for Integrated Protein Science Munich (CIPSM), Department of Pharmacy – Center for Drug Research, Ludwig-Maximilians-Universität München, Munich, Germany.; 7 Division of Ocular Neurodegeneration, Institute for Ophthalmic Research, Centre for Ophthalmology, University of Tübingen, Tübingen, Germany.; 8 Edward S. Harkness Eye Institute, New York-Presbyterian Hospital, New York, New York, USA.; 9 Jonas Children’s Vision Care and Bernard & Shirlee Brown Glaucoma Laboratory, Department of Ophthalmology, Columbia University Medical Center (CUMC), Edward S. Harkness Eye Institute, New York, New York, USA.

**Keywords:** Ophthalmology, Gene therapy, Genetic diseases, Retinopathy

## Abstract

Retinitis pigmentosa (RP) is a major cause of blindness that affects 1.5 million people worldwide. Mutations in cyclic nucleotide-gated channel β 1 (*CNGB1*) cause approximately 4% of autosomal recessive RP. Gene augmentation therapy shows promise for treating inherited retinal degenerations; however, relevant animal models and biomarkers of progression in patients with RP are needed to assess therapeutic outcomes. Here, we evaluated RP patients with *CNGB1* mutations for potential biomarkers of progression and compared human phenotypes with those of mouse and dog models of the disease. Additionally, we used gene augmentation therapy in a CNGβ1-deficient dog model to evaluate potential translation to patients. *CNGB1*-deficient RP patients and mouse and dog models had a similar phenotype characterized by early loss of rod function and slow rod photoreceptor loss with a secondary decline in cone function. Advanced imaging showed promise for evaluating RP progression in human patients, and gene augmentation using adeno-associated virus vectors robustly sustained the rescue of rod function and preserved retinal structure in the dog model. Together, our results reveal an early loss of rod function in *CNGB1-*deficient patients and a wide window for therapeutic intervention. Moreover, the identification of potential biomarkers of outcome measures, availability of relevant animal models, and robust functional rescue from gene augmentation therapy support future work to move *CNGB1*-RP therapies toward clinical trials.

## Introduction

Retinitis pigmentosa (RP) is a genetically heterogeneous cause of blindness, affecting approximately 1 in 4,000 individuals (1). Mutations for nonsyndromic RP have been identified in over 80 different genes (RetNet, Retinal Information Network) (2). The phenotype is characterized initially by decreased rod-mediated function, loss of dim-light vision followed by constriction of peripheral vision, a decrease in visual acuity and, in many instances, progression to legal blindness. Patients develop attenuation of superficial retinal blood vessels, optic disc pallor, and characteristic “bone-spicule” pigment clumping in the midperipheral retina (1). Currently, there is no cure for RP, although gene therapy approaches for other inherited retinal degenerations are showing promising results in phase I and II clinical trials (3–11). The development of therapies for inherited retinal degenerations such as RP is facilitated by relevant animal models that recapitulate the human phenotype and that can be used for preclinical development and refinement of therapy. Prior to clinical trials, it is important to also ascertain whether there are suitable biomarkers of progression of retinal disease that can be used to assess the efficacy of therapy within a practical time frame.

Mutations in the rod cyclic nucleotide-gated channel β 1 (*CNGB1*) gene cause autosomal recessive RP type 45 (RP45) (12–23) (Supplemental Table 1; supplemental material available online with this article; https://doi.org/10.1172/JCI95161DS1) and account for approximately 4% of autosomal recessive RP cases (1). The *CNGB1* gene codes for the β subunit (CNGβ1a) of the rod photoreceptor cyclic nucleotide-gated (CNG) ion channel, with additional splice variants being expressed in the retina (glutamic acid–rich proteins 1 and 2 [GARP1 and GARP2]), olfactory sensory neurons (CNGβ1b), and other tissues (24). Rod photoreceptor CNG channels are nonspecific cation channels in the outer segment cell membrane consisting of 3 cyclic nucleotide-gated channel α 1 (CNGα1) subunits and 1 CNGβ1a subunit (24, 25). CNGβ1a is important for the trafficking of the channel to the outer segment, its positioning within the cell membrane, and control of the channel’s temporal activity (see ref. 26 for a review). Binding of cyclic GMP (cGMP) in the dark results in transient opening of the CNG channel and rod photoreceptor depolarization. With light-induced activation of the phototransduction cascade, there is a reduction in cGMP levels mediated by the G protein activation of a phosphodiesterase (PDE6). Reduced cGMP leads to closure of the channels and hyperpolarization of the rod.

Some phenotyping of patients with RP45 has been previously published and typically describes night blindness from childhood and a later-onset of loss of peripheral vision, leading to an RP diagnosis at around 30 years of age and legal blindness by 60 years of age (12, 13, 16, 18, 22).

A mouse model, *Cngb1-X26*, generated by excising exon 26 and resulting in loss of the full- length CNGβ1a protein, was reported to have a significant decrease in rod function and retinal degeneration (27). Gene augmentation therapy was shown to rescue the phenotype in this model (28).

Recently, we identified a spontaneous mutation in canine *Cngb1* in dogs with recessively inherited progressive retinal degeneration (29). Early loss of rod vision and slow photoreceptor degeneration were observed (29). The canine mutation is downstream of the *Garp* region and results in a lack of full-length *Cngb1a*, thus modeling the majority of reported RP45 cases and closely resembling the *Cngb1-X26* mouse model.

Here, we demonstrate that detailed advanced retinal imaging shows promise with regard to outcome measures for a CNGβ1-RP therapy trial. Moreover, our detailed phenotypic analysis demonstrates that the 2 preclinical models with *Cngb1* mutations recapitulate the human RP45 phenotype. Finally, we report that gene therapy to introduce a normal copy of canine *Cngb1a* into the rod photoreceptors results in robust, sustained restoration of rod function and retinal structural preservation in *Cngb1^–/–^* dogs and represents what we believe to be an important preclinical step toward gene augmentation therapy for human RP45.

## Results

### Human patients with RP45 show early loss of night vision and a slowly progressive photoreceptor degeneration.

Clinical and genetic characteristics of each patient are summarized in Table 1. The cohort (*n* = 8) consists of 4 sporadic cases and 2 sibling pairs, with a mean age of 37.1 years (range, 14–70 years). Five of the patients are of European descent (patients 3–6 and 8) and three are Hispanic (patients 1, 2, and 7). A total of 8 different *CNGB1* mutations were identified. Three of the mutations had been previously described in RP45 patients: c.2284C>T, p.Arg762Cys (16, 30); c.1896C>A, p.Cys632* (15); c.3150delG; and p.Phe1051Leufs*12 (15, 22). Five novel mutations (1 nonsense, 2 frameshift, and 2 intronic/splice) were detected: c.2508C>A, p.Tyr836*; c.522_523insC, p.Lys175Glnfs*4; c.2544_2545insC, p.Leu849Profs*3; c.1122-9G>A; c.2218-2A>G (Table 1 and Supplemental Table 1). The 2 novel intronic mutations were found in patient 4. c.2218-2A>G resides in a canonical splice acceptor site. c1122-9G>A is predicted by Human Splicing Finder software (31) to introduce a strong new splice acceptor site that would insert an additional 7 bp to the 5′ end of exon 15, with a resulting frameshift and predicted premature stop codon. We performed a minigene assay and confirmed the predicted alteration in splicing and the resulting frameshift (Supplemental Figure 1).

**Figure 1:**
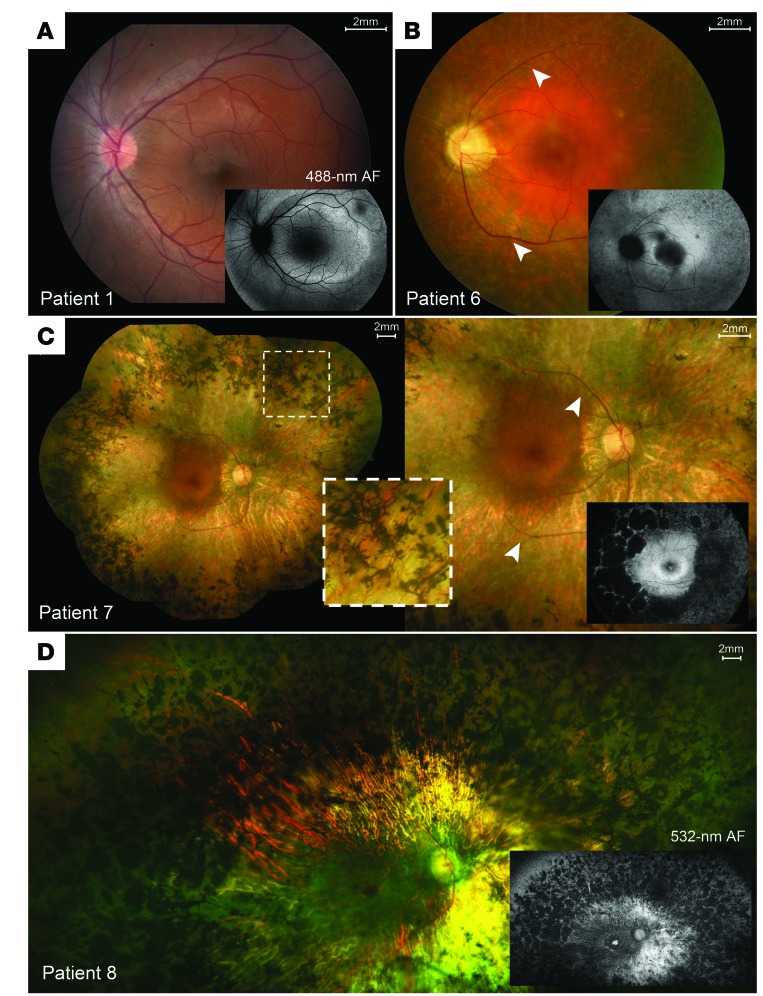
Spectrum of disease severity in patients with *CNGB1*-associated RP. Color fundus montages and corresponding AF images of the left eye of patient 1 (p.Phe1051Leufs*12 homozygous) (**A**), patient 6 (p.Leu849Profs*3; p.Lys175Glnfs*4) (**B**), and the right eyes of patient 7 (p.Cys632*; p.Phe1051Leufs*12) (**C**) and patient 8 (p.Arg762Cys homozygous), illustrating typical presentations of RP features: (**B**–**D**) waxy pallor of the optic disc, severe attenuation of the retinal vasculature (white arrowheads), and bone-spicule pigment clumping in the mid-periphery (insets). (**A**) The left macula of patient 1 (p.Phe1051Leufs*12 *homozygous*) shows largely unremarkable features for retinal degeneration. REC+ thickness is defined as all visiblelayers between the inner nuclear layer-outer nuclear layer (INL/ONL) complex and the Bruch’s membrane-choroidal (BM/Choroid) interface.

**Figure 2:**
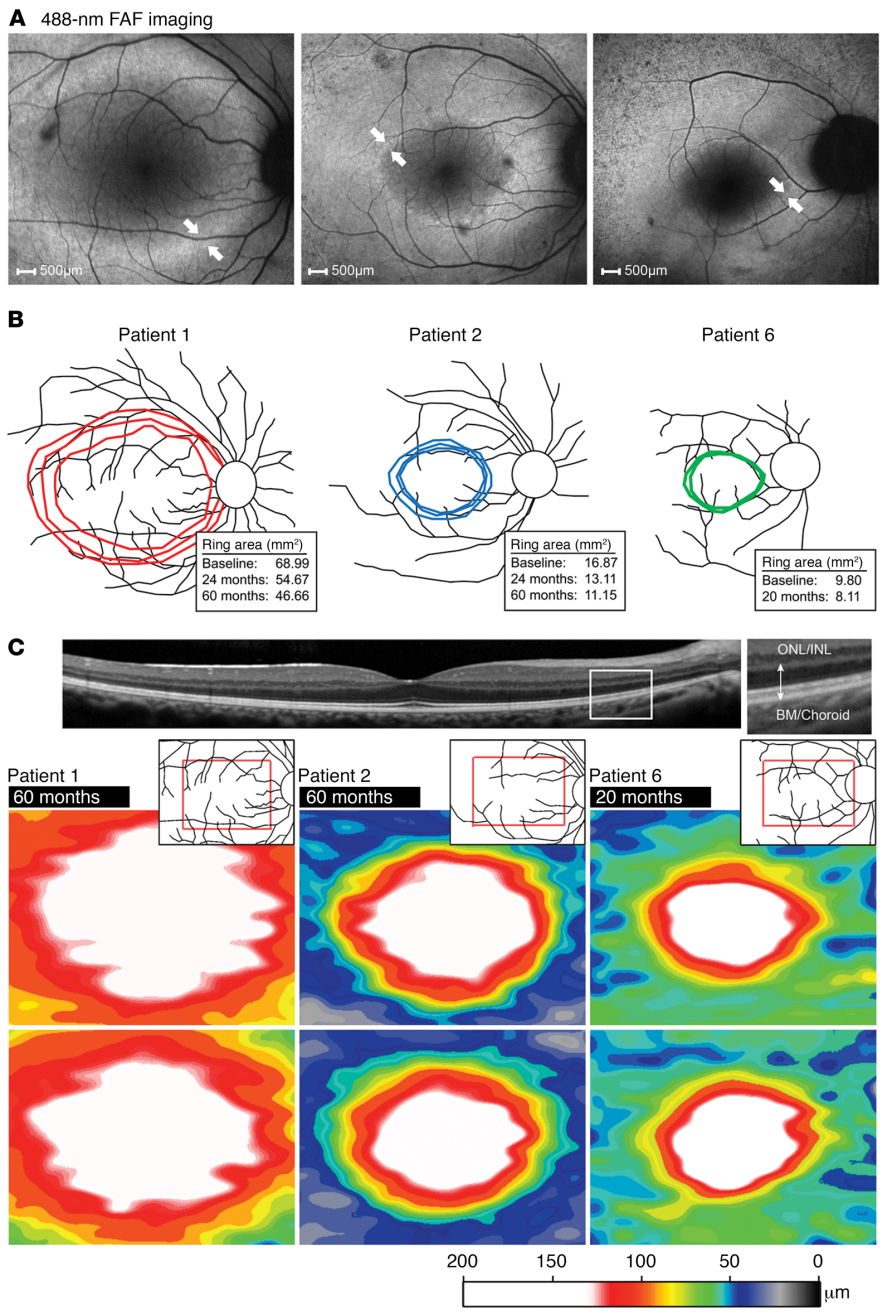
Progressive AF ring constriction and photoreceptor layer thinning in affected patients homozygous and compound heterozygous for *CNGB1* mutations. Affected patients homozygous for *CNGB1*: patients 1 and 2 (p.Phe1051Leufs*12); affected patient heterozygous for *CNGB1*: patient 6 (p.Leu849Profs*3, p.Lys175Glnfs*4). (**A**) FAF imaging (488-nm) of the right eye in each patient revealed the inner and outer border (white arrows) of a progressively constricting region (ring), delineating the centrally preserved area of retinal function. (**B**) Retinal schematic illustrating the constriction size (mm^2^) and shape of the centrally preserved region over various time intervals (insets): after 60 months in patient 1 (red) and patient 2 (blue) and 20 months in patient 6 (green). (**C**) Color-coded maps of total REC^+^ thickness after 60 months in patients 1 and 2 and after 20 months in patient 6 from a segmented macular SD-OCT scan within the position of the retina enclosed in the red rectangle (upper right inset). The right eye of each patient is shown, where white on the color scale (>125 μm) denotes the range of REC^+^ thickness in healthy eyes. REC^+^ thickness is defined as all visible layers between the inner nuclear layer–ONL (INL-ONL) complex and the Bruch’s membrane–choroidal interface (SD-OCT, inset). BM, basement membrane.

All patients had childhood-onset nyctalopia but on presentation had best-corrected visual acuity (BCVA) ranging from 20/20 to 20/100 and remained largely stable over time. Visual fields were constricted to 2 to 18 degrees within the macula. Ophthalmic evaluations of patients 5, 6, 7, and 8 revealed posterior subcapsular cataracts, a waxy pallor appearance of the optic disc, cystoid macular edema, and severe attenuation of the retinal vasculature (Figure 1, B–D, white arrowheads). We observed “bone-spicule” intraretinal pigment migration in the mid-peripheral retina of patients 3–8. Patient 1 (Figure 1A) and patient 2 presented at an earlier disease stage and did not show changes typical of retinal degeneration on fundoscopy. An autofluorescence (AF) ring of variable size (indicative of lipofuscin accumulation) was apparent on fundus autofluorescence (FAF) imaging circumscribing an area of relatively functional retina (Figure 1, A–D, insets, and Figure 2A). The average rate of AF ring constriction varied with size. Patient 1 had the largest ring (68.99 mm^2^ in the right eye at baseline), which progressed at a loss of 14.32 mm^2^ by year 2 and 8.01 mm^2^ by year 5 (Figure 2B and Supplemental Figure 2A). Patients with smaller rings (<10 mm^2^) had constriction at a slower rate: patient 6 had lost 1.69 mm^2^ by year 2. Quantitative thickness maps generated from 19 spectral domain optical coherence tomography (SD-OCT) raster scans of the photoreceptor-attributable layers between the vitread boundary of the outer nuclear layer (ONL) and the Bruch’s membrane–choroidal interface (receptor + [REC^+^] layer) revealed significant thinning in the perifoveal regions over time (Figure 2C). The maps in are scaled according to the reported range of REC^+^ layer thickness in healthy eyes (>150 μm, white color) (32). The respective REC^+^ layer thicknesses in all 3 patients were uniformly decreased in areas closer to the fovea than to the position of ellipsoid zone (EZ) disruption and the border of the AF ring. The extent of thinning progressed gradually from approximately 125 μm (red) to the approximately 60-μm (green) region, where the border of the AF ring was positioned (Figure 2). Profiles of REC^+^ thickness in a single SD-OCT scan in each patient indicated that the observed thinning within the area circumscribed by the AF ring fell below the 95% CIs of healthy, age-matched eyes (Supplemental Figure 2B). ). Each profile also showed significant central thickening of REC^+^ (>95% CIs of healthy eyes) within the central 1-mm diameter of the foveal region. Thickness peaked at the foveal center in all cases and remained stable over time relative to the peripheral thinning near the edge of observable degeneration or the border of the AF ring. Analysis of the of individual SD-OCT scans revealed unusual but consistent characteristics in all patients that included a shallow foveal pit, continuous lamination of the plexiform layers, and widening of the ONL at the center (Supplemental Figure 3).

Full-field electroretinogram (ffERG) testing revealed generalized dysfunction of rods in all patients. Scotopic responses were nonrecordable in the older patients (patients 5–8) and attenuated in the younger patients (patients 1–4). Attenuated 30-Hz flicker and single-flash photopic responses were also evident in the older patients (patients 5–8), while we detected only marginally decreased photopic responses and implicit time delays in the younger patients (patients 1–4) (Table 1 and Supplemental Figure 2C).

### Cngb1^–/–^ mice show a progressive loss of retinal thickness with a slow loss of cones.

**Figure 3:**
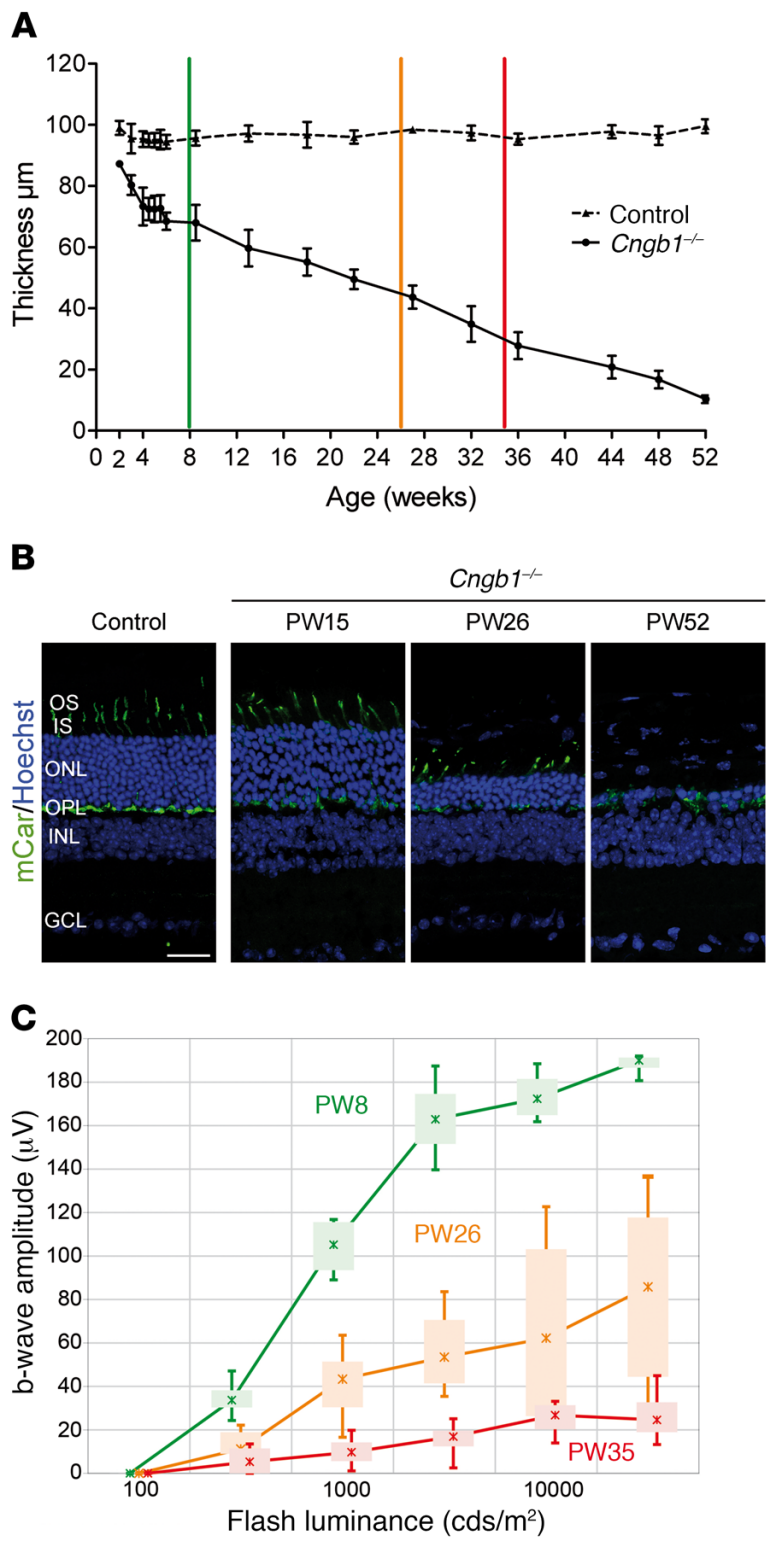
*Cngb1^–/–^* mice show a progressive loss of photoreceptor structure and function. (**A**) Age-related loss of the REC^+^ layer in *Cngb1^–/–^* mice compared with WT mice, as measured by SD-OCT imaging. The colored vertical bars indicate the ages at which photopic ERG b-wave amplitudes were measured in **C** (mean of *n* = 4–6 for each time point). (**B**) IHC with a cone marker (cone arrestin) showing morphologically affected but still-persisting cones after advanced thinning of the ONL (representative images from 3 mice). Scale bar: 25 µm. (**C**) Photopic cone b-wave amplitudes of *Cngb1^–/–^* mice plotted against stimulus strength at 2, 6, and 8 months of age (mean of 4 for each time point). Data represent the mean ± SD. PW8, postnatal week 8; PW26, postnatal week 26; PW35, postnatal week 35.

*Cngb1^–/–^* (*Cngb1-X26*) mice have progressive retinal thinning accompanied by a progressive loss of photoreceptors and their function (Figure 3). We measured the age-related loss of outer retinal thickness (REC^+^ layer) by in vivo cross-sectional SD-OCT imaging (Figure 3A). *Cngb1^–/–^* mice showed a slowly progressive, almost linear thinning of the REC^+^ layer within the observed time frame of 2 to 52 weeks of age. While rod photoreceptors degenerated, we found that cone morphology was also compromised, as indicated by a loss of cone outer segments (Figure 3B). This is in keeping with the progressive loss of cone function during photoreceptor layer thinning that was detected by photopic ERG measurements (Figure 3C).

### Mutation in Cngb1^–/–^ dogs leads to exon skipping and expression of a shortened CNGB1 product.

Reverse transcriptase PCR (RT-PCR) spanning the previously reported site of the exon 26 mutation in canine *Cngb1* (29) (c.2387delA;2389_2390insAGCTAC; Supplemental Figure 4, A and B) and direct Sanger sequencing showed that the mutation caused skipping of exon 26 (data not shown), introducing a premature stop codon early in exon 27 (Supplemental Figure 4C). The truncated product partly escaped nonsense-mediated decay, leading to a relative expression level approximately 40% of that of WT transcript levels in the controls (Supplemental Figure 5A). There was a complete absence of full-length CNGβ1 in the photoreceptors of *Cngb1^–/–^* dogs (29), but IHC using an antibody that targets CNGβ1 between the N-terminal GARP region and the predicted mutation site revealed an accumulation of truncated protein within the inner segments (Supplemental Figure 5B).

### Cngb1^–/–^ dogs have slow photoreceptor loss and relative preservation of cones.

**Figure 4:**
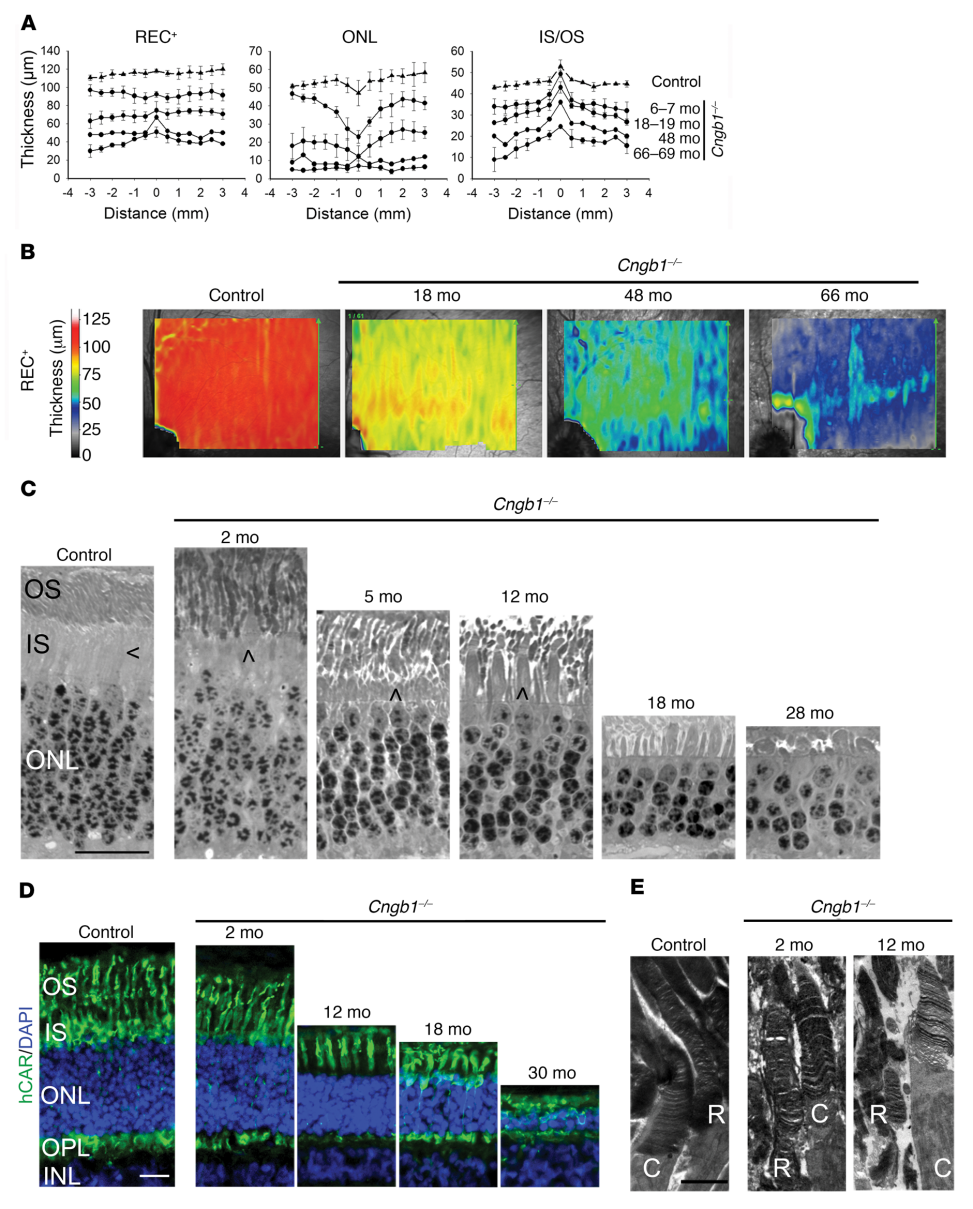
*Cngb1^–/–^* dogs have a progressive retinal thinning with preservation of the REC^+^ in the area centralis. (**A**) Measurement of REC^+^, ONL, and inner segment/outer segment (IS/OS) layer thickness by SD-OCT cross-sectional images in a vertical plane through the area centralis, measured every 0.5 mm. The negative numbers are inferior to the area centralis. Control dogs: *n* = 3; *Cngb1^–/–^* affected dogs: *n* = 3 dogs 6–7 months of age; *n* = 3 dogs 18–19 months of age; *n* = 1 dog 48 months of age; *n* = 2 dogs 66–69 months of age. (**B**) Heatmaps demonstrating preservation of photoreceptor thickness in the area centralis and horizontally along the visual streak. REC^+^ thickness in *Cngb1^–/–^* dogs of different ages compared with a control (WT) dog. *n* = 3 control dogs; *n* = 3 *Cngb1^–/–^* dogs at 18 months of age; *n* = 1 *Cngb1^–/–^* dog at 48 months of age; and *n* = 2 *Cngb1^–/–^* dogs at 66 months of age. (**C**) Representative images of plastic-embedded semi-thin retinal samples from an 8-week-old control dog compared with samples from a *Cngb1^–/–^* dog. The inner segments of cones are located adjacent to the inner segments of rods in the control dog. Shortening of the rod inner segments in the *Cngb1^–/–^* dogs resulted in cone inner segments extending to the level of the rod outer segments. Rod outer segments appeared disorganized and deteriorated over the first 28 months. Initially, cone inner segments appeared grossly normal and then, with rod loss, initially appeared widened (at 12 months) but then became shortened and atrophied (at 28 months). Sections (500-nm) were stained with epoxy tissue stain. Arrows indicate the cone inner segment. Scale bar: 20 μm. *n* = 4 control dogs; *n* = 1 *Cngb1^–/–^* dog at 2, 5, 12, and 28 months of age; *n* = 2 *Cngb1^–/–^* dogs at 18 months of age. (**D**) Representative images of IHC with hCAR (labels the entire length of the cones) show well-preserved cone morphology in the younger animals. In the older *Cngb1^–/–^* affected dogs (18 and 30 months of age), the cones were still visible, albeit shortened. Scale bar: 20 μm. *n* = 2 control dogs; *n* = 2 *Cngb1^–/–^* dogs at 2 and 18 months of age; *n* = 1 *Cngb1^–/–^* dog at 12 and 30 months of age. (**E**) Representative transmission electron microscopic images of rods (R) and cones (C) show a reasonably normal arrangement of rod discs at 2 months of age in the *Cngb1^–/–^* dog, but by 12 months of age, the rod outer segments had deteriorated, but the cone outer segments appeared relatively normal. Scale bar: 2 μm. *n* = 4 controls; *n* = 1 *Cngb1^–/–^* dog at 2 and 12 months of age.

Color fundus imaging revealed an initial hyporeflective appearance of the fundus in the tapetal area, which, with progression, was accompanied by mild attenuation of the superficial retinal vasculature and eventually obvious signs of retinal thinning (tapetal hyperreflectivity; Supplemental Figure 6A). On FAF imaging, a region of AF appeared in the center of the area centralis of a *Cngb1^–/–^* dog at approximately 3 months of age (Supplemental Figure 6B). The area centralis is the canine equivalent of the human macula (33). The earliest change detectable by SD-OCT in *Cngb1^–/–^* puppies was a loss of definition of the zones between the external limiting membrane (ELM) and the interdigitation zone (IZ) in the periphery from 3 months of age. These zones represent the photoreceptor inner and outer segments and interface with the retinal pigment epithelium (RPE). The loss of definition progressed with age, with the central retina remaining unaffected until later in the disease progression (Supplemental Figure 7). We observed slowly progressive thinning of the REC^+^ layer (which represents the entire length of the photoreceptors), with thinning occurring initially in the more peripheral retina, while the area centralis showed relative preservation (Figure 4A). We further examined changes in the thicknesses of the different photoreceptor components that make up the REC^+^ (outer plexiform layer [OPL], ONL, ELM, myoid zone [MZ], EZ, outer segments, IZ, and the RPE–Bruch’s complex) (Supplemental Figure 8) and found that, compared with more peripheral retina, the center of the area centralis actually had an earlier thinning of the ONL but better preservation of the zones representing photoreceptor inner and outer segments, which accounted for the overall preservation of the REC^+^ thickness (Figure 4A). The spatial preservation of REC^+^ not only involves the area centralis but also the visual streak, as shown in the heatmaps in Figure 4B. The visual streak is a horizontal zone of higher photoreceptor density extending temporally and nasally from the area centralis.

Retinal sections showed that there was an early disruption of the normally ordered demarcation between the inner segment and outer segment layers, with cone inner segments extending between the rod outer segments at as early as 2 months of age in the peripheral retina of *Cngb1^–/–^* dogs (Figure 4C). A progressive loss of rows of photoreceptor nuclei occurred, such that by 28 to 30 months of age, only 3 to 4 rows remained in the central retina. We observed a relative preservation of cone photoreceptors (Figure 4, C and D). With disease progression, the cone inner segments became initially broader (by 12 months of age) and then stunted (by 18 and 28 months of age). Transmission electron microscopy showed that in young *Cngb1^–/–^* dogs, the rod outer segments had uniformly stacked discs (e.g., at 8 weeks of age), but with disease progression, they became disorganized, while the morphology of the cone outer segments remained better preserved (Figure 4E).

**Figure 5:**
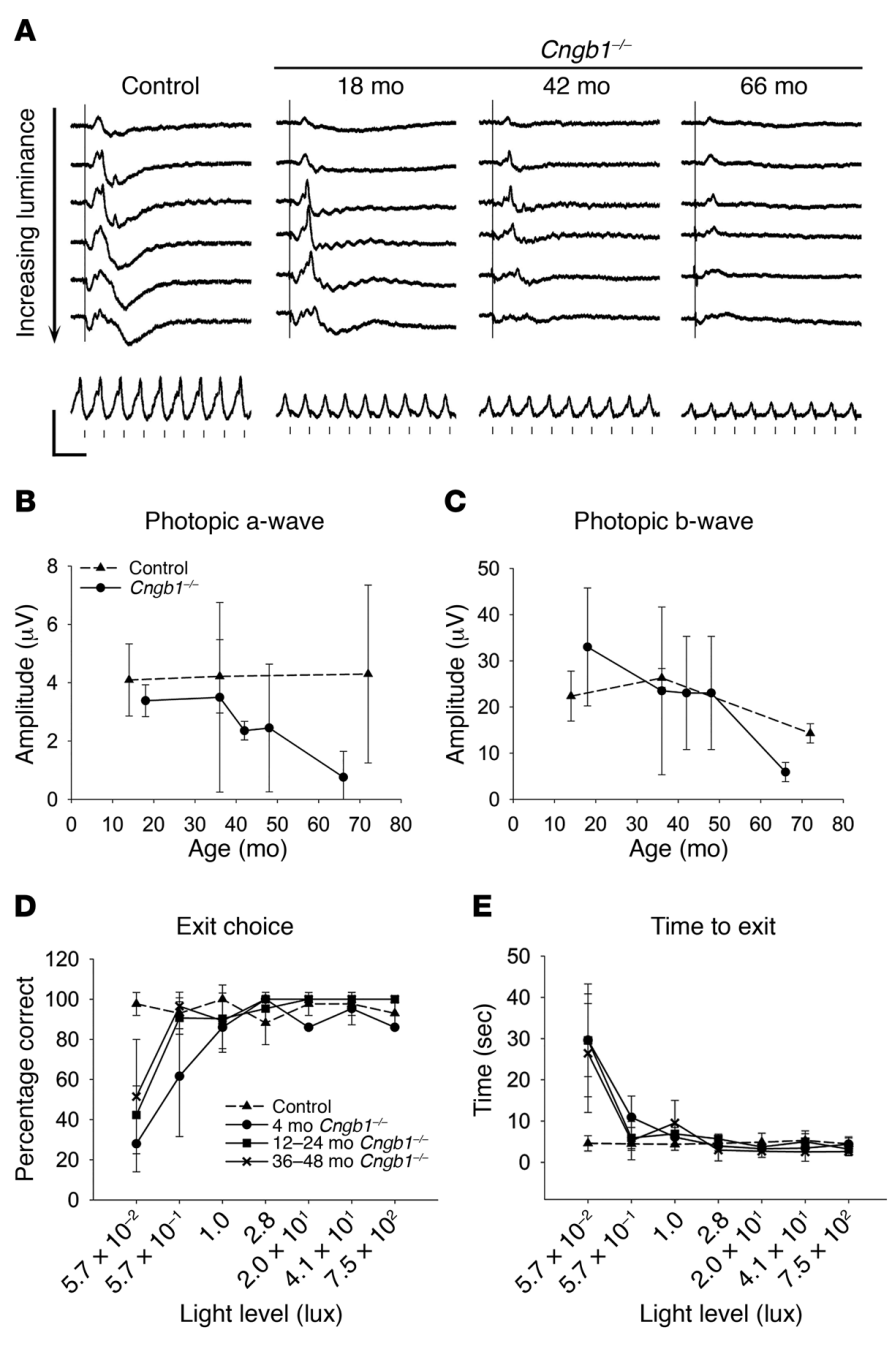
Cone function slowly declines with age in the *Cngb1^–/–^* dog. (**A**) Photopic single-flash ERG tracings in response to the following stimuli in the light-adapted eye present on a background of 30 cd/m^2^: –0.4, 0.0, 0.4, 0.9, 1.4, and 1.9 log cds/m^2^ (top to bottom tracings), and at the bottom, a photopic 33-Hz flicker response at 0.4 log cds/m^2^. (**B** and **C**) Change in the mean (± SD) photopic a-wave (**B**) and b-wave (**C**) amplitudes in response to the 0.4 log cds/m^2^ stimulus with age. The mean photopic a-wave amplitude for *Cngb1^–/–^* dogs was significantly lower at 42 and 66 months of age (*P* < 0.05, Student’s *t* test). The mean photopic b-wave was significantly reduced at 66 months of age (*P* < 0.01, Student’s *t* test). *n* = 2 *Cngb1^–/–^* dogs at each time point; *n* = 3 controls at 14 and 36 months; and *n* = 2 controls at 72 months. (**D** and **E**) Results of vision testing showing the percentage of dogs that made the correct exit choice (**D**) and the time taken to exit (**E**). At all ages tested, the affected dogs had reduced visual function at the lowest light level. Bright light vision was maintained in all age groups tested. Control dogs: *n* = 6; *Cngb1^–/–^* dogs: *n* = 3 for 4-month-old and 12- to 24-month-old dogs and *n* = 4 for 36- to 48-month-old dogs.

Cone function gradually declined with age (Figure 5). However, cone-mediated vision was well preserved, with vision of the *Cngb1^–/–^* dogs being comparable to that of normal control dogs at all lighting levels except the lowest level, which assessed rod vision (Figure 5, D and E).

### Gene augmentation therapy in Cngb1^–/–^ dogs restores the CNG channel in treated retinal regions, resulting in rod function and retinal preservation.

A total of 8 eyes of young *Cngb1^–/–^* dogs (Supplemental Table 2) were used in the gene therapy study. We used an adeno-associated virus vector serotype 5 that delivered canine *Cngb1* under the control of a human *GRK1* promoter (AAV5-*hGRK*-*cCgnb1*) (dose details are provided in Supplemental Table 2). An initial pilot study in 1 *Cngb1^–/–^* dog (14-033, right eye [OD]) resulted in a small improvement in ffERG amplitudes detected 3 months after injection and improved performance in vision testing at the lowest light level (data not shown). A higher dose of the same vector in the second eye of the same dog resulted in a more substantial ERG rescue and vision testing rescue. The repeat administration of vector to dog 14-033 showed no indication of a resulting adverse immune response in clinical, SD-OCT, or IHC studies, and excellent ffERG and vision testing outcomes were achieved in the second eye.

**Figure 6:**
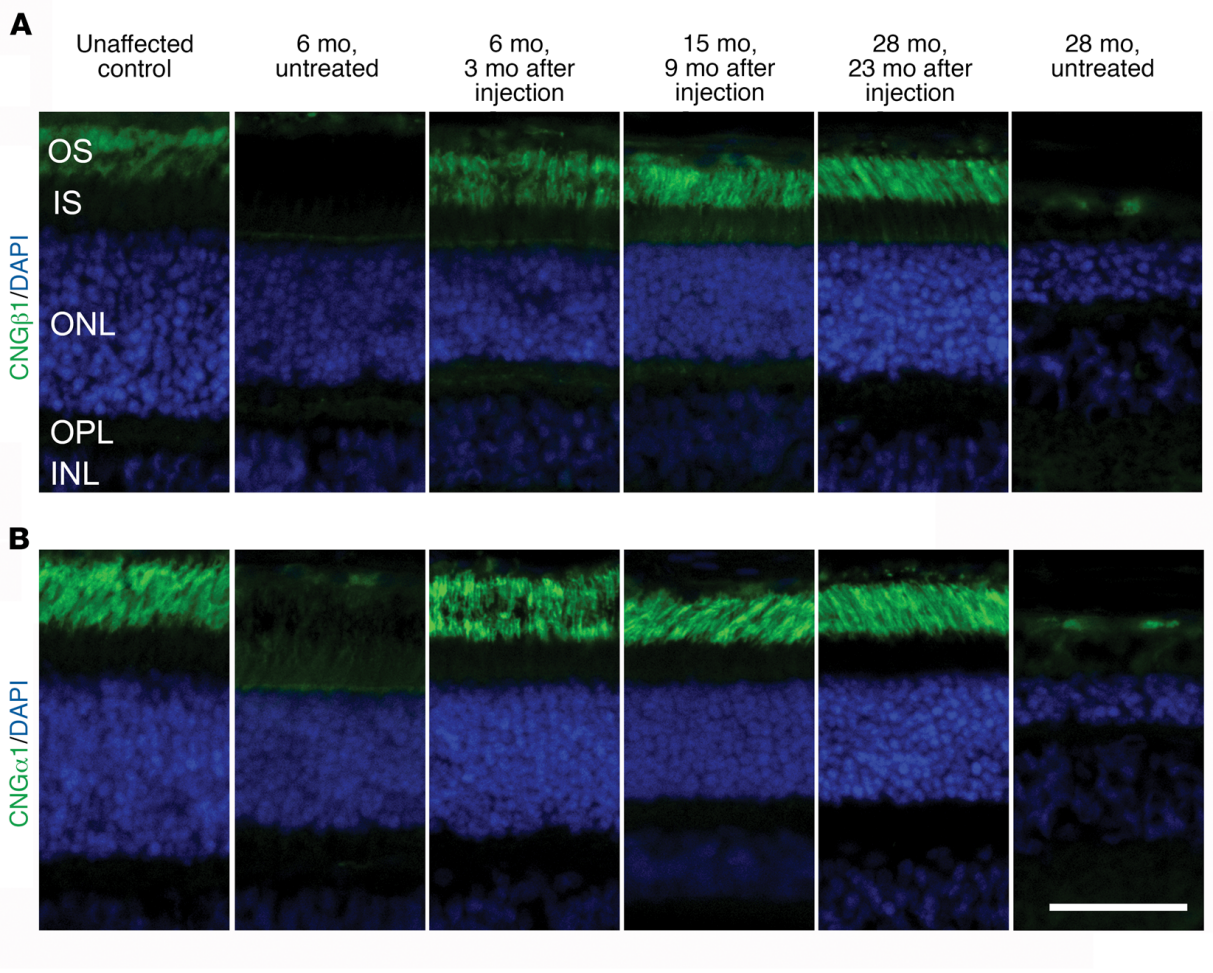
Gene augmentation therapy results in appropriate rod *Cngb1* expression and restores *Cnga1* expression. (**A**) CNGβ1 (green: antibody targeted CNGβ1 distal to the mutation site) was expressed in the outer segments of the treated regions of *Cngb1^–/–^* retinae 3, 9, and 23 months after treatment. The untreated region from the retinae 3 months after injection and the 28-month-old untreated *Cngb1^–/–^* retinae did not express full-length CNGβ1. (**B**) CNG*α*1 (green) was expressed and correctly targeted to the photoreceptor outer segments in the treated regions of *Cngb1^–/–^* retinae 3, 9, and 23 months after treatment, but was not detectable in the untreated region. Scale bar: 50 μm.

**Figure 7:**
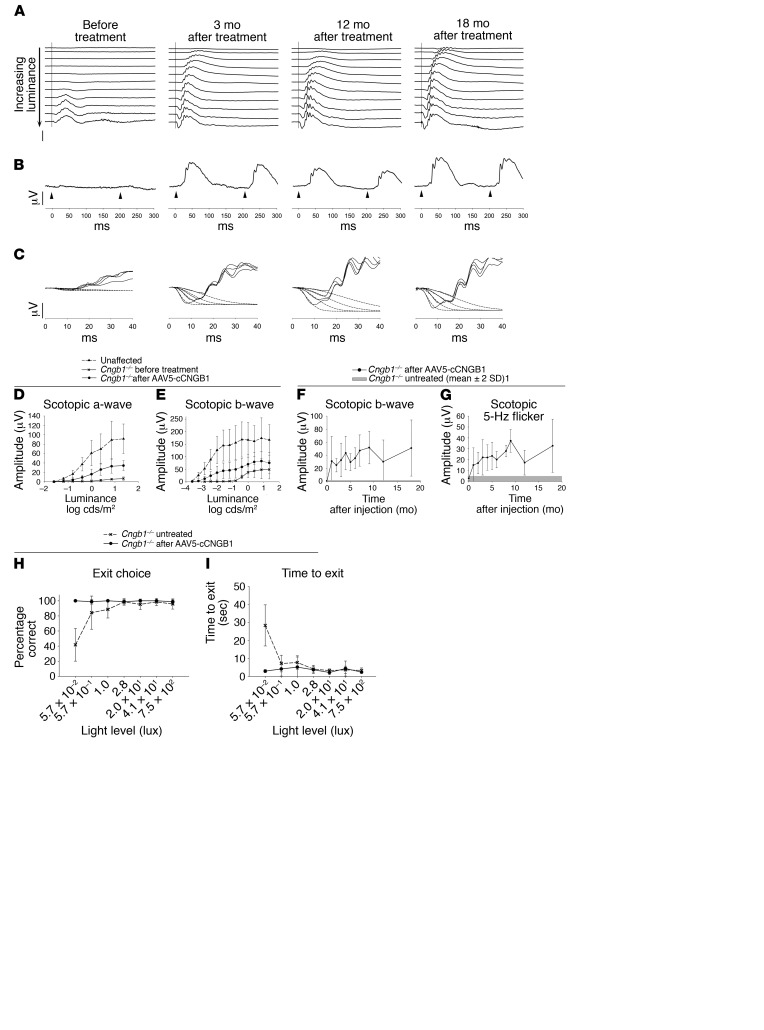
Sustained rescue of rod function by gene therapy. (**A**–**C**) Scotopic ERGs before treatment and 3, 12, and 18 months after subretinal AAV5-*hGRK*1-*cCngb1* treatment (dog 14-055 right eye). (**A**) Luminance response series. Note the obvious lowering of the response threshold and increased a- and b-wave amplitudes (stimulus luminances ranged from –3.7 to 0.4 log cds/m^2^). (**B**) Scotopic 5-Hz flicker responses at –1.6 log cds/m^2^ luminance (vertical scale bars: 50 μV; horizontal scale bars: 50 ms). (**C**) Fit of the leading edge of the dark-adapted a-wave to the Hood and Birch model. The solid lines are the raw ERG data and the dotted lines the derived fits to the leading edge of the a-wave. (**D** and **E**) Mean (± SD) stimulus response ERG plots for scotopic a- and b-waves comparing unaffected control dogs (*n* = 4) with *Cngb1^–/–^* dogs before and 3-months after subretinal AAV5*-hGRK1*-*cCngb1* treatment (*n* = 7). Compared with before treatment, all mean a-wave responses were significantly improved (*P* < 0.05 and *P* < 0.01, 2-tailed, paired Student’s *t* test). The b-wave responses were also significantly improved (*P* < 0.05 to *P* <0.01, 2-tailed, paired Student’s *t* test), with the exception of the responses to the strongest stimuli (*P* = 0.052 and *P* = 0.054, 2-tailed, paired Student’s *t* test). (**F** and **G**) Duration of ERG rescue. Mean ± SD of scotopic b-wave in response to a stimulus of –2 log cds/m^2^ (**F**) and scotopic 5-Hz flicker at –1.6 log cds/m^2^ luminance (**G**), with time after injection. The gray bar represents the mean amplitude of untreated dogs ± 2 SD. Number of treated eyes at each time point: before treatment (0), 1, 2, and 3 months, *n* = 7;4 and 5 months, *n* = 4; 6, 7, and 9 months, *n* = 3; 12 and 18 months, *n* = 2. (**H** and **I**) Vision test results before versus 3 months after treatment. (**H**) Percentage of dogs that made the correct exit choice at each of 7 lighting levels. Pretreatment *Cngb1^–/–^* dogs made more exit choice errors at the dimmer light levels, and these dogs almost always chose correctly 3 months after gene augmentation. The improvement was significant at the lowest light intensity (*P* < 0.0001, 2-tailed, paired Student’s *t* test). (**I**) Time to exit. Prior to treatment, the *Cngb1^–/–^* dogs were slower to exit at the low light levels, and 3 months after treatment, the dogs were faster to exit at the low light levels. The difference was significant at the lowest light level (*P* < 0.001, 2-tailed, paired Student’s *t* test).

An additional 6 eyes (of 4 dogs) were injected at the higher titer. IHC showed that CNGβ1 protein was restored to the rod outer segments only in the region of the subretinal injection (Figure 6A, see also Supplemental Figure 9). Expression of CNGβ1 protein rescued the expression of previously downregulated CNGα1 in the treated regions (Figure 6B) (and not outside of the treated area or in untreated *Cngb1^–/–^* eyes; data not shown and ref. 29), suggesting the formation of functional heterotetrameric CNG channel complexes. Further evidence that the CNG channel subunits had formed a functional channel complex was provided by ffERG and vision testing, both of which showed a dramatic improvement in rod-mediated function (described below). Figure 7A shows the ERG results for dog 14-055 (OD), with a much lowered response threshold and a waveform typical for WT dogs (although approximately one-third the amplitude of breed- and age-matched controls). The rod 5-Hz flicker response prior to treatment was almost nonexistent but was robust following gene therapy (Figure 7B). As a direct assessment of rod photoreceptor phototransduction, we performed fits of the leading edge of the rod a-wave using the Hood and Birch model based on the original Lamb and Pugh model (Figure 7C). These fits showed a significant increase in maximal receptor response (*R_max_*) over pretreatment values (*P* < 0.05, paired *t* test). ERG rescue was maintained in both eyes of dog 14-055 until the last time point assessed (18 months after injection). Plots of the mean a- and b-wave amplitudes against stimulus strength 3 months after treatment showed a substantial improvement in the response threshold of the scotopic ERG (~1 log unit for the a-wave and >2 log units for the b-wave) (Figure 7, D and E) for all eyes treated with the higher dose. The ERG rescue was maintained over the long term, as illustrated in Figure 7, F and G (amplitudes of scotopic ERGs elicited by a flash of low luminance that resulted in a rod response and a 5-Hz flicker response, also indicative of rod responses).

All eyes treated with the higher titer had significantly improved vision at the lowest light level. When using the treated eye (eyes were tested in turn by covering the contralateral eye with an opaque contact lens), the dogs correctly chose the open exit 100% of the time at the lowest light level 3 months after injection compared with a mean of 42% of the time for untreated controls (*P* = 1.3 × 10^–5^). Their exit times were also improved being a mean of 3 seconds 3 months after treatment compared with 28 seconds for the untreated controls (*P* = 1.0 × 10^–4^) (Figure 7, H and I). The improvement in vision testing outcomes was maintained in all treated dogs for the duration of the study (data not shown).

**Figure 8:**
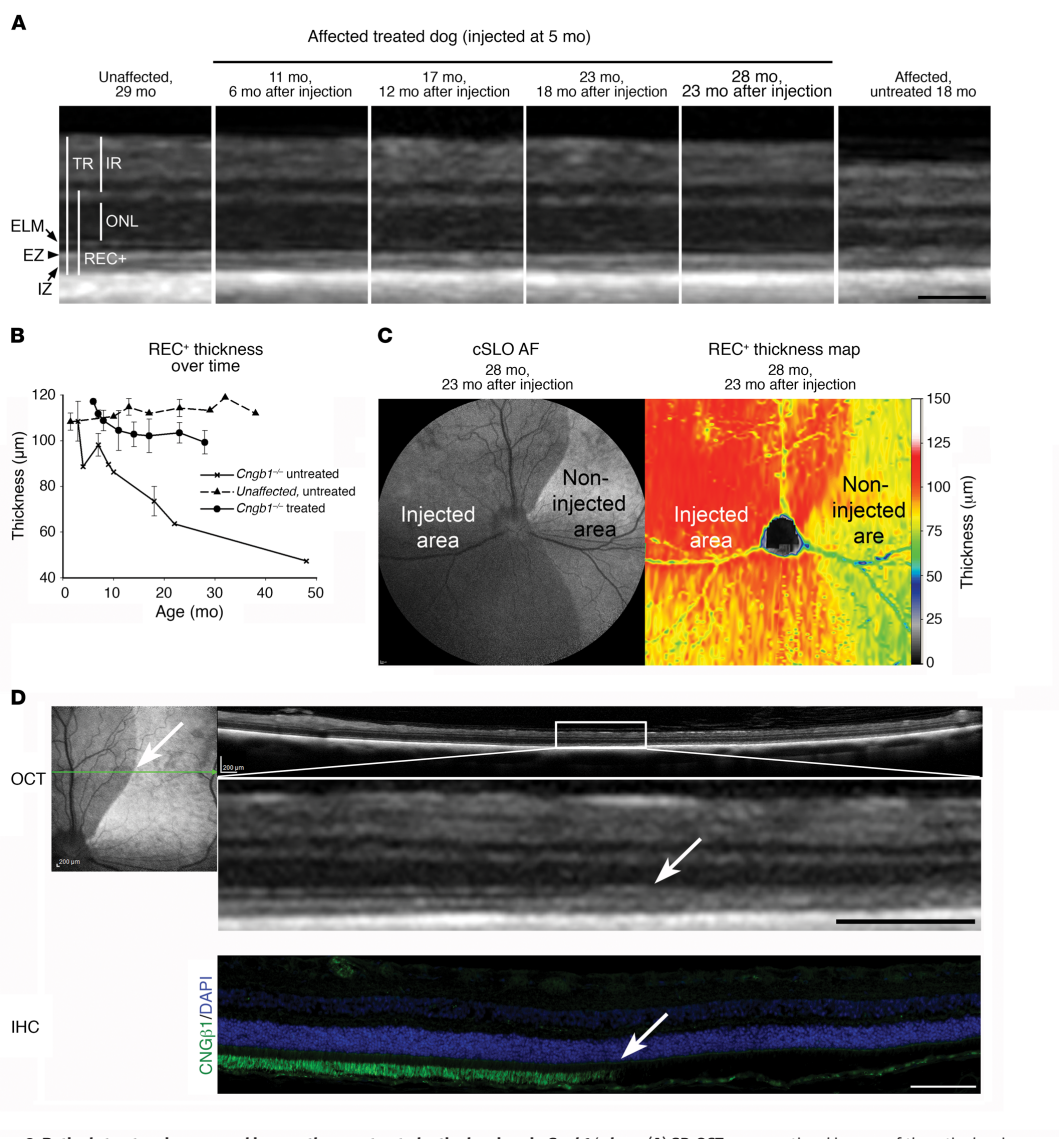
Retinal structure is preserved in gene therapy–treated retinal regions in *Cngb1^–/–^* dogs. (**A**) SD-OCT cross-sectional images of the retinal region of an AAV5-*hGRK1*-*cCgnb1–*treated *Cngb1^–/–^* dog showing preservation of the retinal layers. In addition to ONL preservation, the ELM, EZ, and IZ appear to have better definition compared with untreated *Cngb1^–/–^* dogs. A WT control retina is shown for comparison. IR, inner retina; TR, total retina. (**B**) Plot of the mean thickness of the REC^+^ layer with age in *Cngb1^–/–^* treated retinae (*n* = 2) compared with *Cngb1^–/–^* untreated and unaffected dog retinae. The first time point of the *Cngb1^–/–^* measurement was 1 month after treatment. The untreated *Cngb1^–/–^* retina had a progressive, age-related decline in thickness. The treated eyes showed an initial decline in thickness of the REC^+^, like the untreated eyes, but then plateaued to remain significantly thicker than the REC^+^ layer of the untreated eye (*P* = 0.019, 2-tailed Student’s *t* test, 17–18 months of age). *n* = 3 untreated *Cngb1^–/–^* dogs. Data represent the mean ± SD. (**C**) FAF cSLO imaging of a treated eye 23 months after injection. The noninjected retinal region had a higher level of AF than did the treated (injected) region. Heatmap shows REC^+^ layer thickness preservation in the treated area of the same eye. (**D**) Cross-sectional SD-OCT images across the junction between injected and noninjected areas of the same eye as in **C**, showing a thinning of the ONL in the noninjected area (boundary is indicated by a white arrow). Also note the better definition of the ELM zone, the EZ, and the IZ zone on the image in the injected region. IHC image of the same region shows that *Cngb1* expression stopped abruptly at the edge of the injected area (white arrow). Scale bars: 100 μm (**A**), 200 μm (**D**, top), and 100 μm (**D**, bottom).

In vivo imaging showed a clear preservation of retinal structure and thickness in the treated region (Figure 8, A and D). Assessment of the mean REC^+^ thickness in the treated retinal regions compared with the same retinal regions in untreated *Cngb1^–/–^* dogs showed that treatment preserved REC^+^ thickness (Figure 8, B and C). We observed an initial continuation of REC^+^ thinning for 3 to 4 months after injection, after which it halted, and REC^+^ thickness was maintained and remained significantly thicker than in either the untreated region of the same eyes or in the eyes of untreated*Cngb1^–/–^* dogs (Figure 8B). The junction between the preserved treated retinal region and the adjacent untreated retinal region became more apparent over time. Within the treated area, the definition of the SD-OCT zones representing the photoreceptor inner and outer segments was clearly preserved in the treated areas, whereas in the untreated adjacent retinal areas, the zones could not be discerned (Figure 8, A and D). Dogs maintained for study for longer than 3 months after treatment had less AF in the injected retinal regions compared with the uninjected regions on FAF imaging (Figure 8, C and inset in D).

## Discussion

Humans with *CNGB1*-related RP (12, 13, 16, 23), mice (*Cngb1-X26*) (27), and *Cngb1*^–/–^ dogs with mutations that spare expression of the alternatively expressed GARP subunits share a similar phenotype characterized by a lack of rod-mediated retinal function from an early age, followed by a slowly progressive age-related loss of cone function and, in humans, constriction of visual fields.

Preservation of the macula is a feature of this (Figure 2) and other forms of RP. The presence of an area centralis in the dog, with its similarity to the human macula, allows the study of changes in a region of higher photoreceptor density, which is an advantage over laboratory rodents, which do not have a comparable retinal region. Like the finding of macular preservation in RP patients, *Cngb1^–/–^* dogs also showed a preserved REC^+^ thickness in the area centralis and visual streak (Figure 4, A and B). The packing density of photoreceptors is highest in these regions, with peak cone density in the center of the region approaching that of the human macula (33). Measurement of the individual SD-OCT–detectable retinal layers that comprise the REC^+^ (OPL, ONL, ELM, MZ, EZ, outer segments, IZ, and RPE–Bruch’s complex) (34) revealed that, unlike in the macula of human patients, there was an early loss of ONL thickness indicating an early loss of photoreceptor nuclei in the *Cngb1^–/–^* dog area centralis. The thickness of the REC^+^ layer was preserved as a result of maintenance of the SD-OCT–detectable zones representing the photoreceptor inner and outer segments and the interdigitation with the RPE. In the central part of the area centralis of the normal dog retina, the number of cones peaks, such that the numbers of rows of cone and rod nuclei are similar (33). Despite this higher number of cones, SD-OCT imaging shows a slight thinning of the ONL layer of the area centralis in normal dogs (33) (also see Supplemental Figure 7, bottom). As occurs in the equivalent human foveal region, the inner segments of canine cones in this region are thinner and elongated (33, 35). It is conceivable that the early ONL thinning in this region in the *Cngb1^–/–^* dog is accounted for by loss of central rods and that the high proportion of cones with elongated inner and outer segments, compared with regions outside the area centralis, accounts for the maintenance of the thickness of the corresponding SD-OCT layers and preservation of REC^+^ thickness in this region. Preservation of cones until later in the disease process may also explain the maintenance of EZ integrity in the area centralis. The EZ is believed to correspond with the distal end of the photoreceptor inner segments, perhaps reflecting the large number of mitochondria in this part of the photoreceptor (36). Loss of intensity or integrity of the EZ has been associated with deterioration of vision in humans with various retinal conditions (reviewed in ref. 37).

As is typical for RP, humans with RP45 show a hyperautofluorescent macular ring on FAF imaging. This is suggested to represent an abnormal perifoveal accumulation of lipofuscin in the RPE associated with disease-related increased photoreceptor outer segment loss (38, 39). In our study, changes in the width of the hyperautofluorescence ring was detectable in patients who underwent serial imaging, with differences being detectable over a period as short as 2 years (Figure 2). Likewise, the degree of thinning of photoreceptor-attributable layers (REC^+^) on SD-OCT progressed within the same follow-up interval, however, thinning appeared to spatially precede the ring constriction in the patients observed in our study. These findings corroborate those of another study describing analogous thinning as well as a functional decline within the apparently unaffected area within the AF ring (38). The ring itself may therefore not be the earliest marker of photoreceptor dysfunction in the retinae of RP patients; however, a direct correlation with structural changes in the outer retina and a consistent pattern of progression have been extensively demonstrated in heterogeneous RP cohorts (40–42). Therefore, although these morphological features may individually be valid biomarkers of disease progression, they are likely reflective of different degenerative processes, particularly as both are derived from separate imaging modalities (32, 43). A more in-depth understanding of the relationship between the morphological features seen on AF (488-nm excitation), which derive principally from the distribution of RPE lipofuscin, and photoreceptor loss is warranted. Nevertheless, these features appeared to change rapidly enough in the patients in this study to suggest that they could be used as a plausible measure of therapeutic outcome for a future clinical therapy trial, although a more thorough assessment of the natural history in a significantly larger cohort of patients with RP45 is needed to confirm this finding.

Consistent with the clinical characterization of RP, the *CNGB1*-RP patients in this study maintained a central area of relatively preserved visual function and acuity; however, this region was structurally abnormal, in that we detected a pattern of REC^+^ thickness that was significantly elevated above the normal range in healthy retinae. Interestingly, visual examination of this region on SD-OCT revealed shallower foveal depressions, an incomplete extrusion of the plexiform layers, and central vertical widening of the ONL in varying degrees across all patients (Supplemental Figure 3). These changes bore an apparent resemblance to foveal hyploplasia (44), though this is a developmental anomaly most often associated with X-linked ocular albinism (45) and has not been described previously as a clinical manifestation of RP. The underlying cause of this apparent thickening of the ONL may be biologically intrinsic to the pathogenesis of CNGβ1 pathogenesis, or perhaps a mechanical stretching of the inner retina due to the deterioration of the outer retinal layers, namely the EZ, as has been previously described in other RP patients (46).

*Cngb1^–/–^* dogs had a pattern of hyperautofluorescence that was different from that of the human patients, showing a small region of brighter AF developing in young *Cngb1^–/–^* dogs in the center of the area centralis and then along the visual streak, with disease progression (Supplemental Figure 6B). The difference in AF patterns between dogs and humans may reflect a species difference in the course of photoreceptor loss between different retinal regions. It is of interest that in gene therapy–treated dogs, by several months after treatment, we observed less AF in the treated region compared with that detected in the untreated region (Figure 8, C and D). This might reflect the reduction in photoreceptor death in the treated regions.

A possible histological explanation for the early loss of definition of the zones representing the photoreceptor inner and outer segments in the peripheral retina on SD-OCT imaging of *Cngb1^–/–^* dogs was the finding on semi-thin sections of a disruption of the usually sharp demarcation between photoreceptor inner segments and outer segment layers, with rod outer segments appearing alongside cone inner segments (Figure 4, C, D, and E). With the progression and loss of the rods, the remaining cone inner segments became wider in diameter and then progressively more stunted.

Photopic (cone-mediated) vision in *Cngb1^–/–^* dogs was well maintained (up to at least 66 months of age), despite the diminishing cone ERG amplitudes. It has been our experience that very few remaining functional cones are required in dog RP models for them to negotiate around obstacles in bright light conditions, and this degree of vision can still be the case even after cone ERG responses can no longer be detected (S.M. Petersen-Jones, unpublished observations).

Currently, there is no definitive treatment for RP. We hypothesized that the *Cngb1^–/–^* dog would be a good candidate for gene augmentation therapy because of the early loss of rod function but only a slow physical loss of rod photoreceptors. IHC showed that this therapy resulted in *Cngb1* expression in outer segments at the earliest time point assessed (3 months after injection), and this was maintained for at least 23 months after injection, but only in the treated retinal regions (Figure 6A and Figure 8D, bottom). We found that the expression of *Cnga1* was also increased, but only in the treated regions, confirming that CNGβ1 is required for the trafficking of CNGα1 to the rod outer segments (Figure 6B). Untreated *Cngb1^–/–^* dog retinae lacked expression of full-length *Cngb1* and had very reduced CNGα1 levels. The labeling of CNG subunits on IHC after gene therapy appeared to be similar to that in normal dogs. Therapy resulted in a dramatic and sustained improvement in rod-mediated ERG responses and in visual function at dim light levels.

This preliminary proof-of-concept study suggested that there was a dose effect: the first eye injected using a lower dose of vector showed less evidence of rescue than did eyes subsequently treated at a higher dose. A dose escalation study is required to establish the optimal dose for the greatest rescue without toxicity. We detected variations in the degree of ERG rescue among eyes, with several possible reasons including individual (biological) variation, age at injection, and the area of retina treated and thus the number of transduced rods contributing to the ffERG. Similar volumes of vector were injected, but, as is typical for subretinal injections, the extent of the retinal detachment (and thus the area treated) varied among eyes.

One dog (both eyes treated) maintained for longer-term study had sustained ERG rescue up to 18 months after injection (the last time point recorded), with no diminution. SD-OCT clearly showed an improvement in the treated area in the appearance of the zones that represent the inner and outer segments of the photoreceptors. We found that the EZ and IZ were clearly visible in the treated area, but their integrity was lost outside of the treated region. SD-OCT measurements of the REC^+^ layer showed that, in the initial few months after treatment, some progressive thinning occurred but was halted and that REC^+^ thickness was subsequently conserved in the treated retinal regions but not in untreated areas, where degeneration occurred to an extent similar to that seen in untreated control *Cngb1^–/–^* dogs. We confirmed the preservation of photoreceptors in the treated regions by retinal histology. The initial loss of REC^+^ thickness in the period following subretinal injection may be due to a combination of the time it takes for transgene expression to become established and the loss of rods that were either not transduced, expressed inadequate levels of CNGβ1, or were already irreversibly on the path to cell death. Loss of photoreceptors in *Cngb1^–/–^* dogs and mice occurs more slowly than it does in some other RP models (e.g., those with phosphodiesterase 6 mutations [ref. 47]), and for this reason, treated animals need to be maintained for adequate periods of time after initiation of therapy to allow for assessment of the structural preservation resulting from therapy.

These promising initial gene therapy results support further investigation of the long-term rescue of rod function and maintenance of retinal structure. Optimization of the vector, both in terms of the specific vector construct and optimal vector dose, is also warranted. Furthermore, in planning for the potential future treatment of human patients, it will be important to study the efficacy of therapy applied at different disease stages. In the current study, younger dogs were treated prior to major loss of rod photoreceptors.

Treatment in humans should, at the very least, be aimed at preserving cone function. Many forms of RP result from mutations in genes expressed in rod photoreceptors, leading to rod photoreceptor death, which is followed by an inevitable secondary loss of cones. Gene augmentation for the rod-led diseases will only be successful while there are still sufficient rods remaining to support continued cone survival. Thus, this requires early diagnosis and treatment. With RP45, the presence of nyctalopia in childhood offers the opportunity for early identification of patients who are suitable for gene augmentation, while there is still a population of potentially rescuable rods.

In summary, we show that patients with mutations in *CNGB1* have night blindness from childhood, with a slow loss of photoreceptors. Their phenotype is similar to that of the *Cngb1-X26* mouse and the *Cngb1^–/–^* dog. Our preliminary study of gene augmentation therapy in *Cngb1^–/–^* dogs showed a robust improvement in rod function, with evidence of photoreceptor preservation and improvement in photoreceptor morphology. The slow loss of photoreceptors, despite an early absence of night vision, provides a potentially large therapeutic window of opportunity. This finding, coupled with the successful gene therapy reported in the *Cngb1-X26* mouse (28) and now the *Cngb1^–/–^* dog, make this an attractive form of RP for gene therapy. Potential outcome measures for human patients could include improvement in rod-mediated ERG responses and rod vision in younger patients, who have a sufficient population of rods available to rescue and contribute to a rod ERG, and halting of progressive changes in the hyperautofluorescence zone and thickness of REC^+^ as indicators of structural preservation.

## Methods

### Human patients

Complete ophthalmic examinations included slit-lamp and dilated fundus examinations. Vision was assessed by the measurement of BCVA (Snellen).

SD-OCT scans and corresponding infrared reflectance fundus images were acquired using a Spectralis HRA+OCT (Heidelberg Engineering). FAF images were obtained using the Heidelberg Retina Angiograph 2 confocal scanning laser ophthalmoscope (cSLO) (Heidelberg Engineering,). The images were acquired by illuminating the fundus with an argon laser source (488-nm excitation) and viewing the resultant fluorescence through a band-pass filter with a short wavelength cutoff at 495 nm. Ultra-widefield AF (532-nm excitation) and color images for patient 8 were acquired with an Optos 200Tx cSLO (Optos PLC). Color fundus photos were obtained with a FF450^plus^ Fundus Camera (Carl Zeiss Meditec). Retinal image analyses were carried out using Heidelberg Explorer software (HEYEX). Constriction of the hyperautofluorescence ring on FAF images was assessed by comparing the difference in areas (mm^2^) within the external ring border between baseline and subsequent examinations. REC^+^ thickness was assessed in single 9-mm and cube (20 × 15/6.2 mm × 4.6 mm, 19 b-scans) high-resolution (HR) SD-OCT scans through the fovea and over the macula, respectively, using HEYEX segmentation software. Two groups of age-matched (20–30 years and 30–40 years) healthy controls were measured and 95% CIs calculated for comparison.

ffERGs were recorded with the Diagnosys Espion Electrophysiology System (Diagnosys) using standard techniques of the International Society for Clinical Electrophysiology of Vision (ISCEV) (48).

Genetic screenings in all patients were carried out at molecular diagnostics laboratories approved by the Clinical Laboratory Improvement Amendments (CLIA). Patients 1 and 2 were tested under the National Ophthalmic Disease Genotyping Network (eyeGENE), and direct sequencing of exon 31 of the *CNGB1* gene was performed. Patients 3 and 5 were first tested by the Asper Ophthalmics (Estonia) Autosomal Recessive Retinitis Pigmentosa Arrayed Primer Extension (APEX) microarray chip (594 mutations on 19 genes) and then by the Massachusetts Eye and Ear Ocular Genomic Institute (Boston, Massachusetts, USA). Patient 4 was tested with Retinitis Pigmentosa Tier 1 Panel screening by the Casey Eye Institute Molecular Diagnostic Laboratory (Portland, Oregon, USA). Segregation in 3 unaffected siblings and both parents of patient 4 was consistent with biallelic inheritance, with only the proband harboring both *CNGB1* variants. Patients 3, 5, and 6 were tested at the Massachusetts Eye and Ear Ocular Genomic Institute using Sanger sequencing of all *CNGB1* exons (ABI BIG DYE chemistry and ABI 3100 automated sequencer) and also using Retinitis Pigmentosa Tier 1 Panel screening performed by the Casey Eye Institute Molecular Diagnostic Laboratory. Parents’ samples were tested for all 3 patients, which was consistent with biallelic inheritance of the variant in each respective patient. Patients 7 and 8 underwent whole-exome sequencing at the Columbia University Laboratory for Personalized Genomic Medicine using the Agilent SureSelectXT Human All Exon V5+UTRs capture method and Illumina HiSeq 2500 sequencing technology. Pathogenicity of found variants data were analyzed using NextGENe software (SoftGenetics). The variant number is in accordance with the NCBI’s GenBank NM_001297.4 (with cDNA numbering starting with the A of the ATG start codon) and NP_001288.3. Variants were considered novel if not previously reported and not present in the NCBI’s dbSNP (http://ncbi.nlm.nih.gov/projects/SNP/), Exome Variant Server (EVS; http://evs.gs.washington.edu/EVS/), or the Exome Aggregation (ExAC) database (http://exac.broadinstitute.org). Splice prediction was performed using the Human Splicing Finder (http://www.umd.be/HSF3/) (31). A minigene assay was performed to investigate a novel splice variant in patient 4 (protocol is found in the Supplemental Methods).

### Animal studies

#### Mice.

*Cngb1-X26* mice as previously described (27) (http://www.informatics.jax.org/allele/MGI:3527896) were used in this study. Control experiments were conducted using mice with the same genetic background. The mouse line was on a mixed background of 129/SvJ and C57-BL6/N, without the *rd8* (*Crb1*) mutation.

#### Dogs.

The dogs used in this study were maintained in a colony at the Michigan State University Comparative Ophthalmology Laboratory. Dogs with a spontaneously occurring mutation in canine *Cngb1* were identified and bred with laboratory beagles to create a breeding colony in which the *Cngb1* mutation segregated the providing dogs that were either homozygous or heterozygous for the mutant allele or homozygous for the WT allele (29).

### Retinal RT-PCR

RT-PCR of canine retinal tissues was performed using standard protocols. Expression levels of retinal transcripts were normalized to succinate dehydrogenase (*Sdha*) mRNA levels. Following RNA extraction using an RNeasy Mini Kit (QIAGEN) and a cDNA synthesis (3′ RACE Kit (Invitrogen, Thermo Fisher Scientific) according to the manufacturers’ instructions, the following mRNA levels were analyzed via real time quantitative PCR (Applied Biosystems StepOne Fast Machine): *Cngb1* (*Cngb1* forward: GGACATCACCGTGTTCCAG; *Cngb1* reverse: TGTCCATCTTAAAGCGACGAG); *Cnga1* (*Cnga1* forward: TCCCAATGTGATTGTTCCAG; *Cnga1* reverse: TCAAACATGGAGGCACTGTC); *Pde6a* (*Pde6a* forward: CCACGTGAAGTGTGACAATG; *Cnga1* reverse: AGCTCTCCTTGCAGGATCTC); and *Sdha* (*Sdha* forward: CGGTCCATGACTCTGGAAAT; *Sdha* reverse: GCAACTGCAGGTACACATGG).

To investigate the effect of the mutation in canine *Cngb1* on splicing, retinal cDNA was amplified using primers flanking canine *Cngb1* exon 26 (forward primer: AGGGTTTTCCCAGTCACGACCGGCCTACCTGCTCTACAGT; this primer included a tag to allow quantification if required; reverse primer: ACCAGGTCTTGACACGGTTC). Gel-purified amplicons were sent to Michigan State University’s Research Technology Support Facility for Sanger dideoxy sequencing on an ABI 3730 Genetic Analyzer (Applied Biosystems).

### Animal ERG

ERGs in mice were performed as previously described (49, 50). ERGs in dogs were recorded as previously described (51, 52). Modeling of the leading edge of the dark-adapted a-wave was performed as described by Hood and Birch (53, 54).

### Animal retinal imaging

SD-OCT of mice was performed as previously described (28, 55), using an adapted Spectralis HRA+OCT system (Heidelberg Engineering) in combination with optic lenses. OCT scans were conducted with a 12° circular scan mode centered at the optic nerve head. Quantification of the REC^+^ layer was performed as described previously (55).

Wide-field color fundus images of dogs were captured using a RetCam II Video Fundus Camera (Clarity Medical). cSLO imaging and SD-OCT of dogs were performed using a Spectralis OCT+HRA. High-resolution cross-sectional images were obtained by line and volume scanning.

### Vision testing of dogs

Vision testing of dogs was performed using a previously described 4-choice vision-testing device (56, 57). Briefly, dogs were placed in the central box of the vision-testing device from which there were 4 tunnel exits. The far ends of 3 of the exits were blocked, and 1 was left open. The open exit was randomly chosen for each run. Performance was tested under 7 lighting settings, ranging from bright to dim light levels. The time to exit the device and the first exit chosen were recorded. The mean correct choice and time to exit were calculated over 14 runs per light intensity. Vision in each eye of the gene therapy–treated dogs was tested separately by placing an opaque contact lens to block vision from the contralateral eye.

### IHC

Mouse eyes were processed for IHC as previously described (28). Vertical cryosections (10-μm) were stained using a rabbit anti-mouse cone arrestin antibody (1:300) (58) (a gift of Wolfgang Baehr, University of Utah School of Medicine, Salt Lake City, UT, USA).

Canine eyes were processed for frozen IHC following previously described methods (29, 59). The following primary antibodies were used: rabbit polyclonal anti-CNGβ1 targeting the C-terminus (downstream of the canine mutation site) (1:500) (HPA039159; Sigma-Aldrich); rabbit polyclonal anti-CNGB1, generated to amino acids 574–763 (60), which is downstream of the GARP region and upstream of the canine *Cngb1* mutation (1:2,000) (FPc21K, a gift of Frank Müller, Institute of Complex Systems, Cellular Biophysics, ICS-4, Forschungszentrum Jülich, Germany); mouse monoclonal anti-CNGα1 (1:10) (a gift of Bob Molday, University of British Columbia, Vancouver, British Columbia, Canada) (61); mouse monoclonal anti-rhodopsin (MS1233PABX, rhodopsin Ab-1, RetP1; Thermo Fisher Scientific); and anti-human cone arrestin (hCAR) (1:10,000) (a gift of Cheryl Craft, Keck Medical School of University of Southern California & USC Roski Eye Institute, Los Angeles, California) (62).

### Plastic-embedded histological sectioning and electron microscopy

Canine eyes for plastic-embedded sectioning and transmission electron microscopy were fixed as previously described (47). Regional retinal sections were dissected, embedded in agarose, post-fixed in 2% osmium tetroxide for 15 minutes, dehydrated in acetone, and then infused with Spurr resin (63). Semi-thin sections (500-nm) were stained with epoxy tissue stain (Electron Microscopy Sciences), and thin sections (70- to 100-nm) were captured on copper grids and stained with 4% uranyl acetate and then Reynolds lead citrate. Semi-thin sections were imaged on a light microscope (Nikon Eclipse 80i; Nikon Instruments). Thin sections were imaged on a JEOL 100CX transmission electron microscope with a Gatan ORIUS camera.

### Gene therapy

The recombinant AAV2/5 vector with the canine *Cngb1* cDNA controlled by a human G protein–coupled receptor kinase 1 (*GRK1*) promoter (AAV5-*hGRK1*-*cCngb1*) (Supplemental Figure 10) was manufactured using previously published methods (64). AAV5-*hGRK1*-*cCngb1* was delivered subretinally into 8 eyes of 5 dogs as previously described (65). Supplemental Table 2 provides details on the dogs used for gene therapy in the study.

### Statistics

*P* values were calculated using the statistical software SigmaPlot, version 12 (Systsat Software). Data were analyzed for normality and equal variance. If the data passed normality and variance tests, a Student’s *t* test was applied. Mean ERG values and vision testing before and after treatment were determined using a paired, 2-tailed Student’s *t* test. Nonparametric data were analyzed using a nonparametric *t* test (unequal variance) or a Mann-Whitney *U* sum test (not normally distributed). A *P* value of less than 0.05 was considered statistically significant.

### Study approval

Patients 1, 2, 7, and 8 were recruited at the CUMC and provided informed consent prior to their enrollment under the IRB-approved protocol AAAI9906 of Columbia University. Patients 3 and 4 were recruited at WEH and provided informed consent under the IRB-approved protocol 17-616EE of WEH. Patients 5 and 6 were examined at WEH and also at the CUMC. All study procedures adhered to the tenets established by the Declaration of Helsinki.

All procedures involving mice were performed with the permission of the Regierung von Oberbayern (Government of Upper Bavaria)and those involving dogs were approved by the IACUC of Michigan State University. All animal experimentation was conducted in accordance with the Association for Research in Vision and Ophthalmology Statement for the Use of Animals in Ophthalmic and Vision Research.

## Author contributions

SMPJ designed and implemented the dog studies and wrote the manuscript. LMO implemented the dog studies and acquired and analyzed data. PAW conducted dog experiments and analyzed results. WL performed studies of human subjects, analyzed data, and wrote the manuscript. JRS analyzed human patients’ FAF results. MT analyzed human patients’ results. SLB was responsible for gene therapy experimental design and vector design. VC produced viral vectors. JEC conducted genetic assessment of patients. EB designed and completed the minigene assay. CS implemented and interpreted mouse experiments. MWS implemented and interpreted mouse experiments. AVL conducted studies of patients and analyzed human data. SM designed and interpreted mouse experiments and wrote the manuscript. WWH designed and developed viral vectors and edited the manuscript. SHT coordinated and analyzed human patient data.

## Supplementary Material

Supplemental data

## Figures and Tables

**Table 1:**
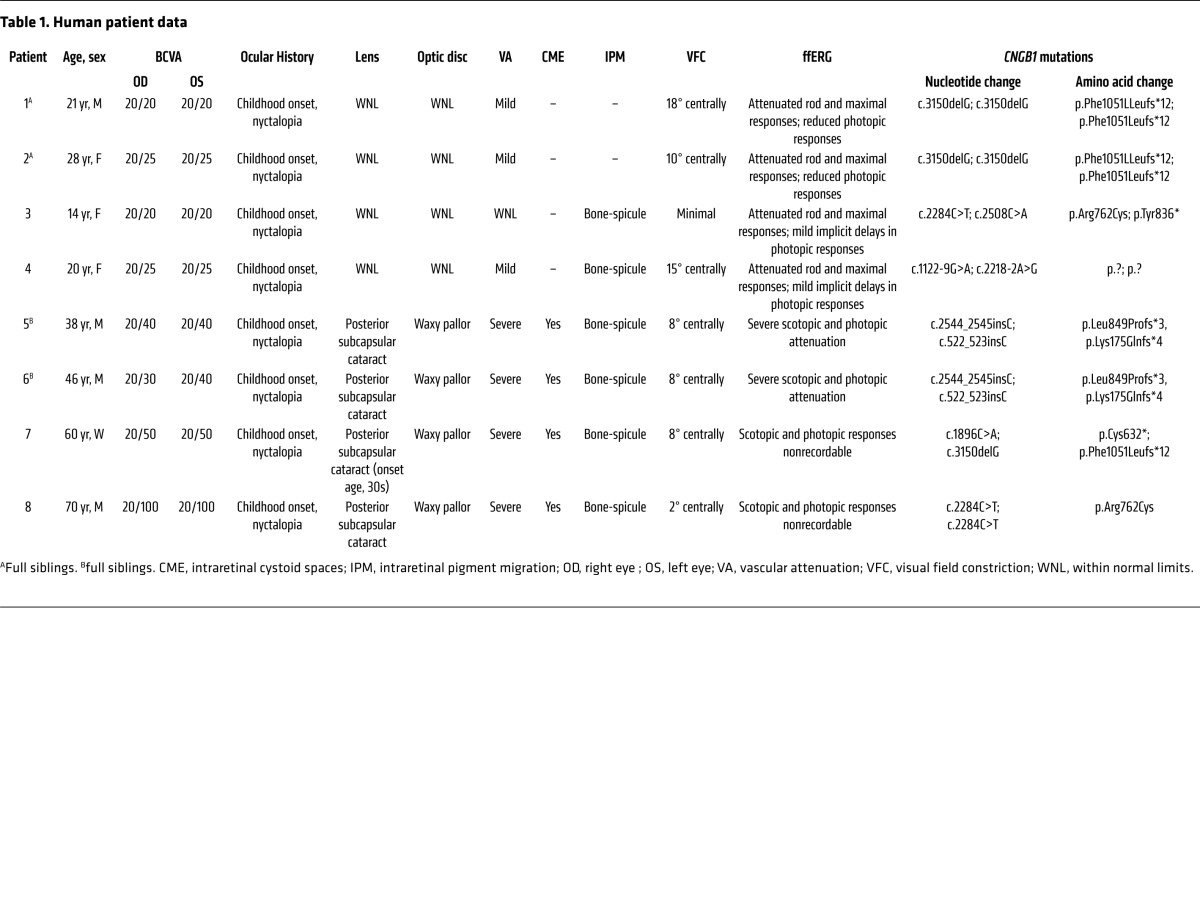
Human patient data

## Version 1 11/20/2017

Electronic publication

## Version 2 01/02/2018

Print issue publication
