# Supplementary material for: Patients and animal models of CNGβ1-deficient retinitis pigmentosa support gene augmentation approach
Source: J Clin Invest. 2017 Nov 20;128(1):190–206. doi: 10.1172/JCI95161 (PMC5749539; doi:10.1172/JCI95161)
Supplement: Supplemental data [file jci-128-95161-s001.pdf]

Supplemental Data

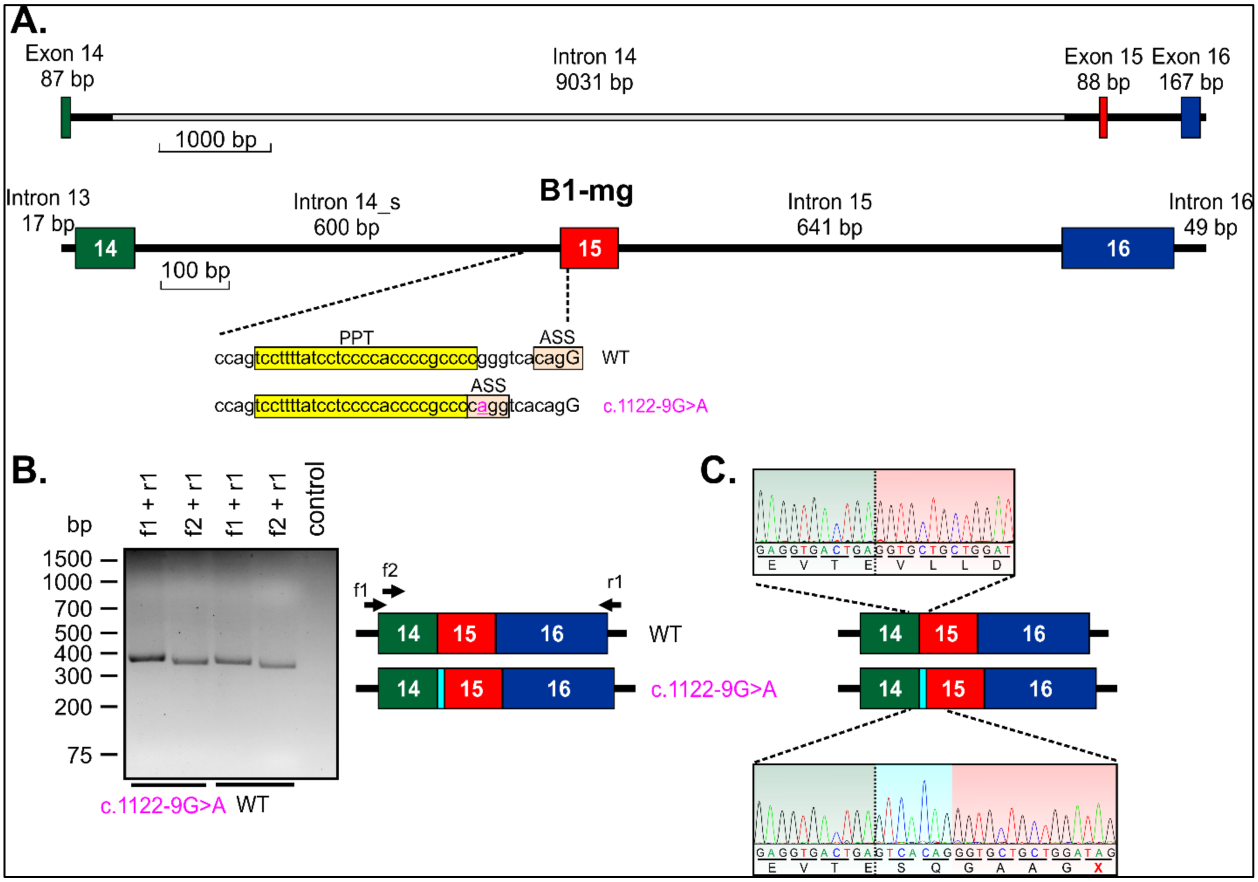

Supplemental Figure 1. *CNGB1* minigene splice assay. (**A**) Top panel: schematic to scale overview of the genomic *CNGB1* structure encompassing exons 14-16 and the flanking intronic sequences. Bp, base pairs. The gray box represents the part of intron 14 that was deleted in the *CNGB1* minigene (B1-mg) shown in the lower panel. The shortened intron 14 (Intron 14_s) contains 300 bp of the native sequences flanking exon 14 and 15, respectively. Dashed lines symbolize the schematic magnification of the 3' acceptor splice site (ASS, red shaded) of exon 15 including the polypyrimidine tract (PPT, yellow shaded) region of wild type (WT) and the c.1122-9G>A mutant as indicated. Note that usage of the novel ASS generated by the c.1122-9G>A mutant results in a 7 bp retention of intron 14. (**B**) Representative reverse transcriptase PCR (RT-PCR) from HEK293 cells transiently transfected with WT or c.1122-9G>A mutant. For

both, WT and c.1122-9G>A, two different primer combinations were used to amplify exons 14-16. The splice product schemes for WT and the c.254-649T>G mutant are shown in the right panel. The 7 bp intron 14 retention for the c.1122-9G>A mutant is symbolized by a cyan box. The first primer combination (f1 + r1) results in a 357 bp PCR product for WT and 364 bp product for the c.1122-9G>A mutant, respectively. By contrast, f2 + r1 primer combination leads to a 337 bp and 344 bp for WT and c.1122-9G>A, respectively. As control, RT-PCR from non-transfected HEK293 cells was performed using f1, f2 and r1 primers. For reproducibility, the HEK293 cell transfection and subsequent RT-PCR experiments were repeated once. (**C**) Representative sequencing results of the exon 14/exon 15 border for WT and c.1122-9G>A as indicated. The c.1122-9G>A mutant causes a frameshift resulting in a stop codon 7 aa downstream of exon 15.

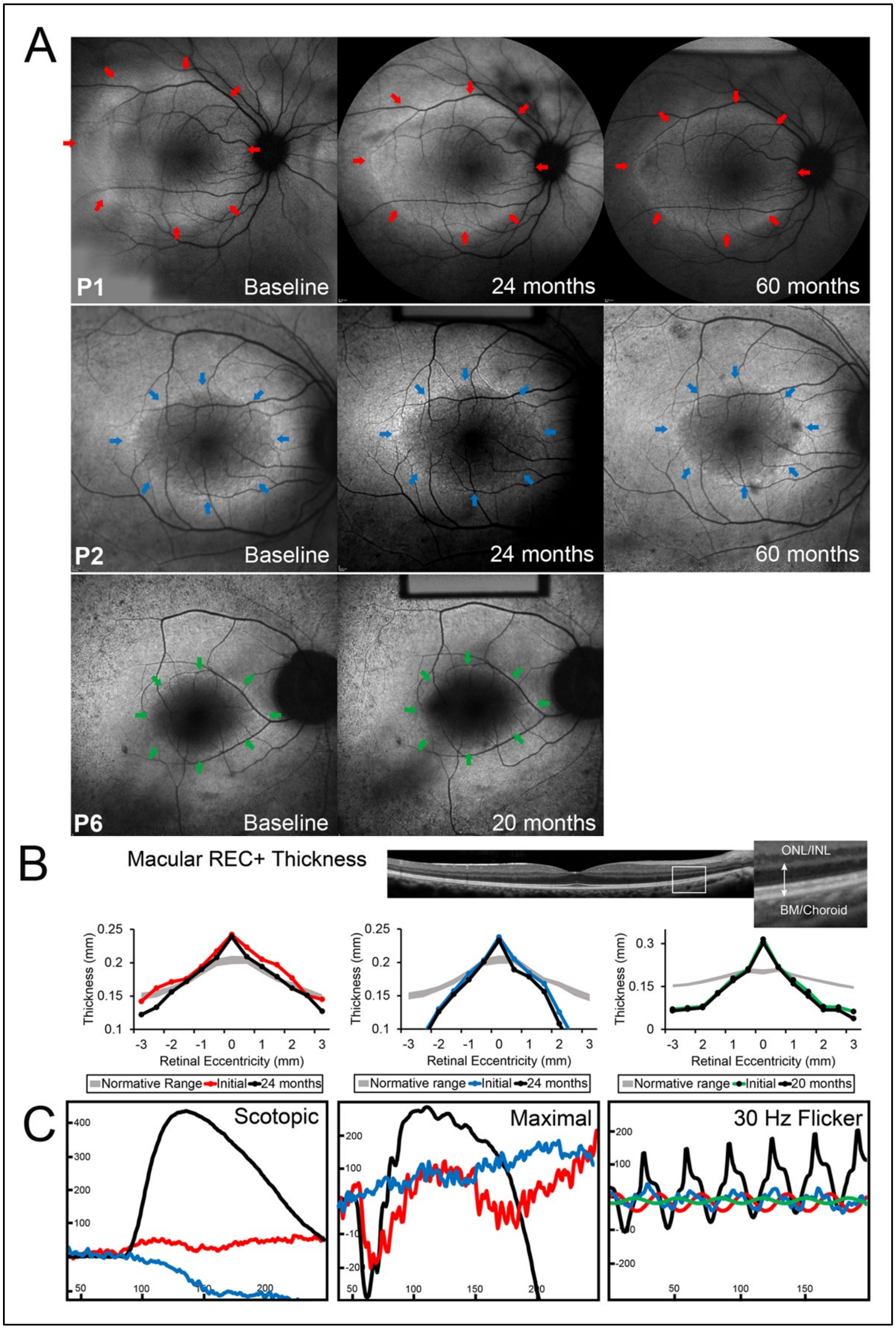

A
P1
Baseline
24 months
60 months
P2
Baseline
24 months
60 months
P6
Baseline
20 months
B
Macular REC+ Thickness
ONL/INL
BM/Choroid
Thickness (mm)
Retinal Eccentricity (mm)
Normative Range
Initial
24 months
Normative range
Initial
24 months
Normative range
Initial
20 months
C
Scotopic
Maximal
30 Hz Flicker

Supplemental Figure 2. (**A**) Serial autofluorescence (AF) imaging in the right eye of Patient 1, Patient 2 and Patient 6. Colored arrows mark the outer border of the constricting autofluorescent ring over time. (**B**) Plot of total receptor+ (REC+) thickness after 24 months in Patient 1 (red) and Patient 2 (blue) and 20 months in Patient 6 (green) in a horizontal spectral domain-optical coherence tomography (SD-OCT) scan through the fovea. The right eye of each patient is shown where 0 in retinal eccentricity denotes the position of the fovea with increasing values oriented in the nasal direction. The shaded gray region in the plot represents the 95% confidence interval of age-matched healthy eyes (n=12, 20-30 years; n=12, 30-40 years). REC+ thickness is defined as all visible layers between the inner nuclear layer/outer nuclear layer (INL/ONL) complex and the Bruch's membrane/choroidal interface in mm (inset). (**C**) Average full-field ERG recordings in each patient (colors ascribed as above) were acquired according to International Society for Clinical Electrophysiology standards. Each patient exhibited extinguished rod function and generalized attenuation and delay of cone function as compared to the waveform recorded from a healthy eye in black.

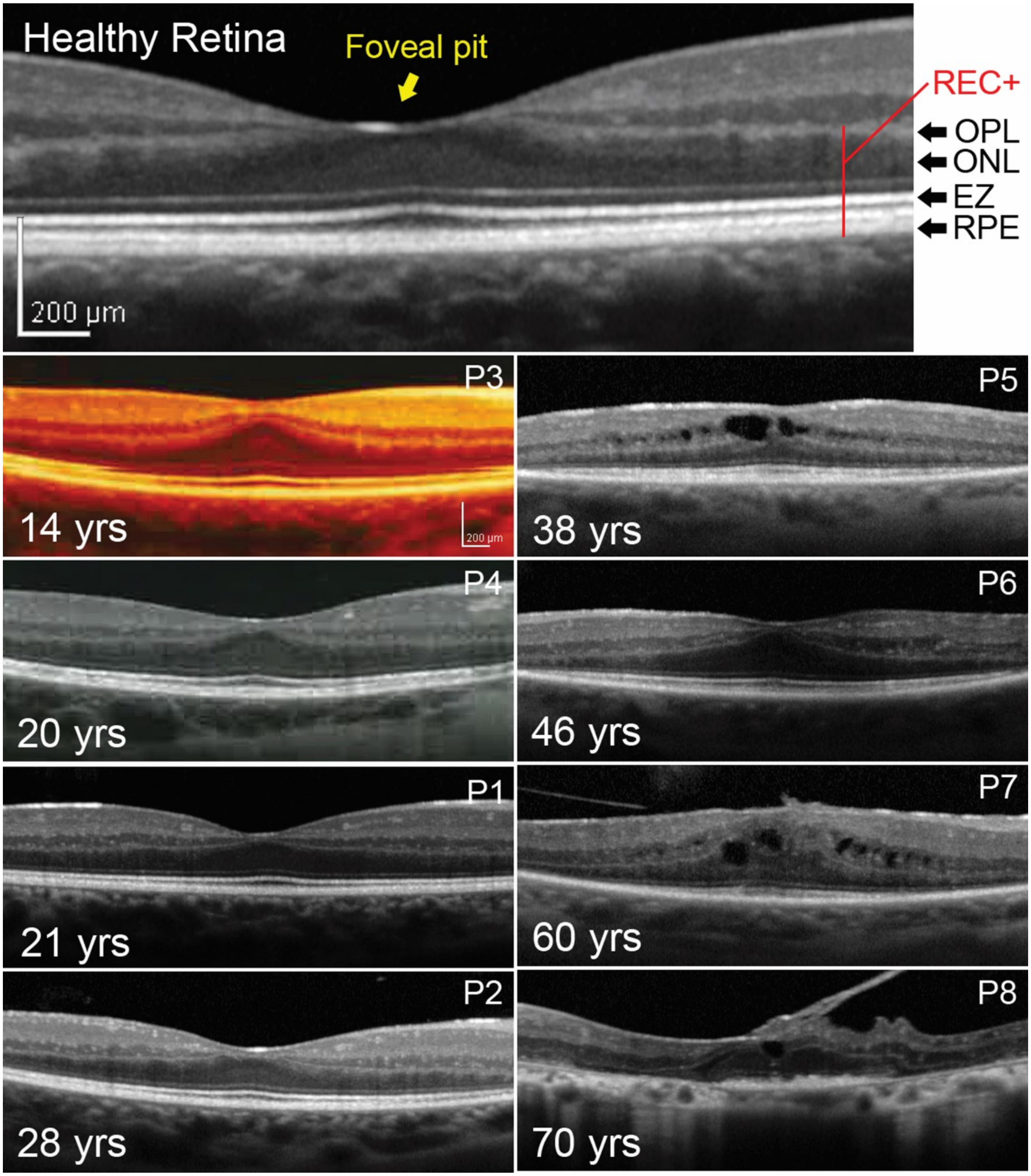

Supplemental Figure 3. Spectral domain-optical coherence tomography scan through the fovea of a healthy retina (top panel) and CNGB1-RP patients. The central fovea (yellow arrow) in a healthy retina is characterized by a deep anatomical depression and the extrusion or displacement of the plexiform layers of the inner retina. CNGB1-RP patients exhibited, in

varying degrees, a comparatively shallower foveal pit, an apparent continuation of the plexiform layers and a thickening of the outer nuclear layer (ONL). Layers attributable to the photoreceptor, termed receptor+ (REC+), consist of the retinal pigment epithelium (RPE), ellipsoid zone (EZ), ONL and outer plexiform layers (OPL). Note the presence of cystoid macular edema in P5-P8. P7 and P8 also exhibit epiretinal membranes.

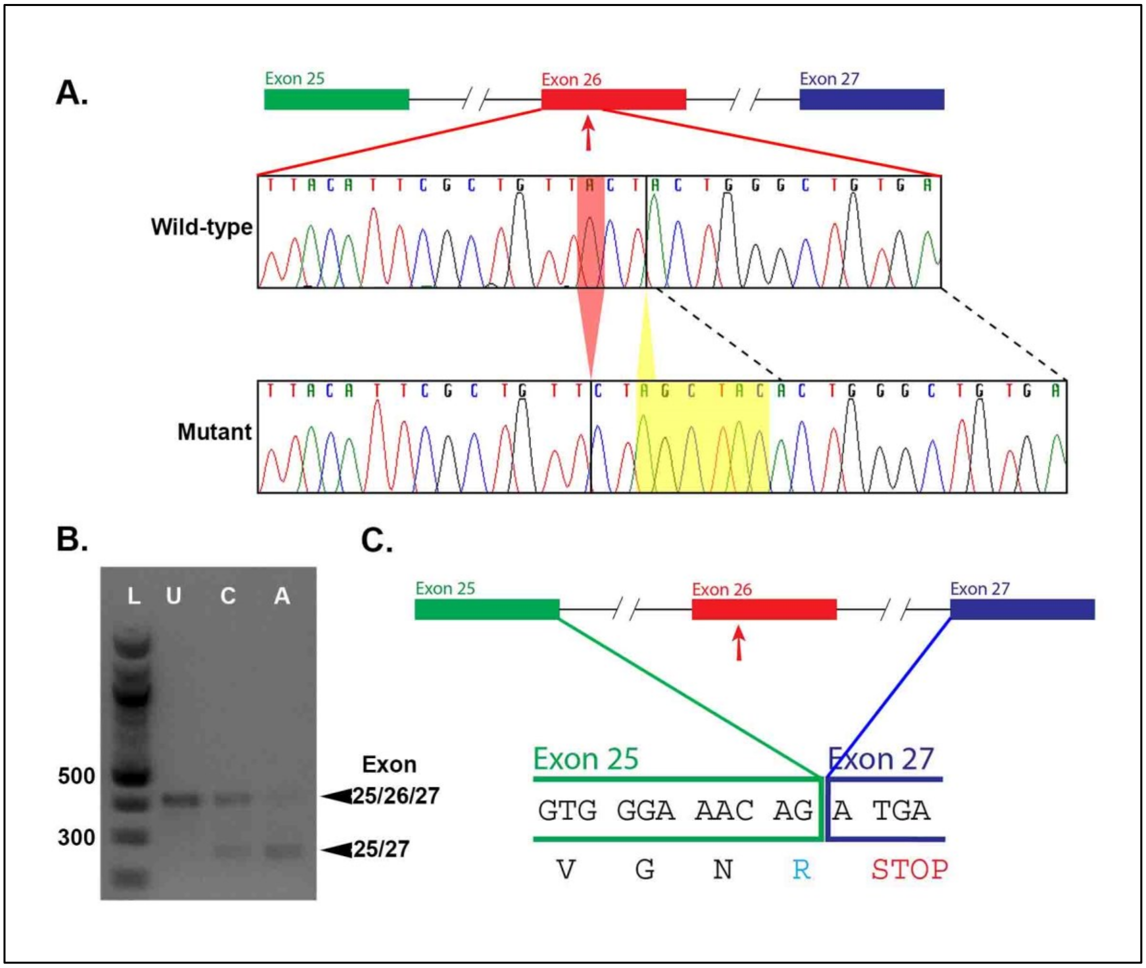

Supplemental Figure 4. The mutation in *Cngb1*$^{-/-}$ dogs leads to skipping of exon 26 and a premature stop codon. (**A**) The mutation in exon 26 of canine *Cngb1* – c.2387delA;2389_2390insAGCTAC. (**B**) Agarose gel showing RT-PCR from retina using primers spanning from exon 25 to exon 27 to *Cngb1*. L = ladder. U = wild-type dog showing a product of expected size (403bp). C= dog heterozygous for the *Cngb1* mutation showing an additional shorter PCR product (266bp). A = dog homozygous for *Cngb1* mutation, the majority of the RT-PCR product is the shorter form. Sanger sequencing showed that the upper product represented normal splicing joining exons 25/26/27. The lower product (25/27) represented a product with exon 26 skipped. (**C**) The effect of exon 26 skipping on predicted coding. A stop codon is predicted early after the frameshift induced by skipping of exon 26.

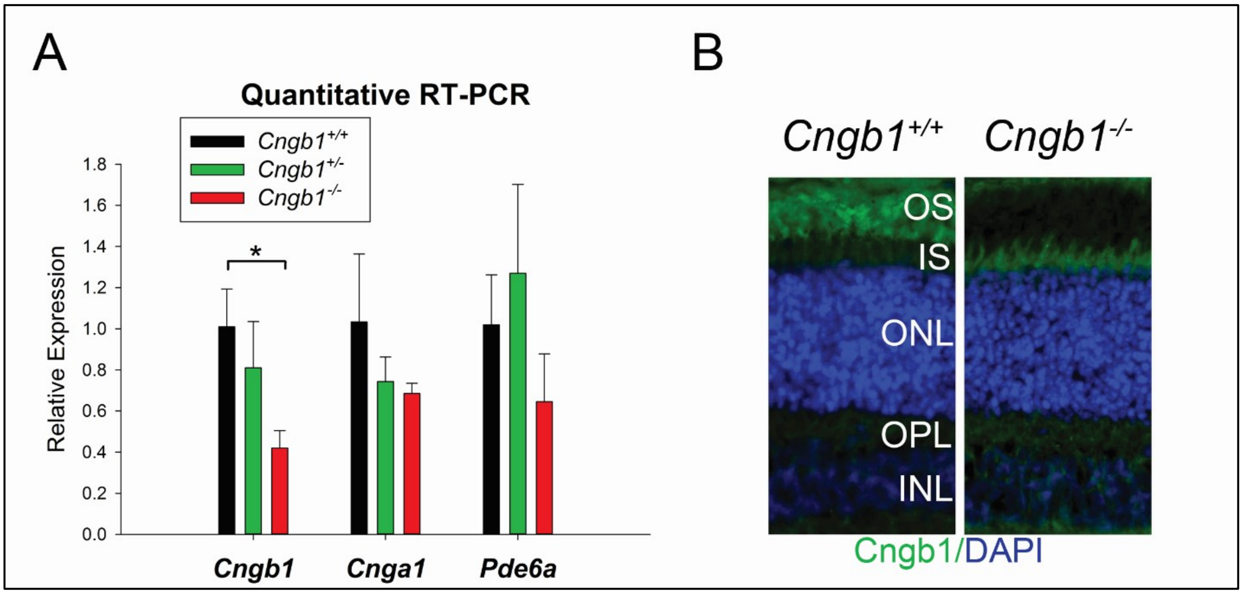

Supplemental Figure 5. **A truncated Cngb1 product is expressed in *Cngb1$^{-/-}$* dogs.** (**A**) Quantitative RT-PCR of retinal mRNA from *Cngb1$^{+/+}$, Cngb1$^{+/-}$* and *Cngb1$^{-/-}$* dogs (mean +/-SD). The results are shown normalized to the level in *Cngb1$^{+/+}$* dogs. The level of *Cngb1* expression in the mutant dogs is about 40% that of wild-type using primers to amplify cDNA upstream of the predicted truncation site. Levels of *Cnga1* and *Pde6a* were not significantly different from wild-type levels. (**B**) Immunohistochemistry using an antibody that targets CNGB1 (residues 574-763) which is transcribed from the region of the gene upstream of the premature stop codon. In the control unaffected dog retina this antibody labels CNGB1 in the rod outer segments. In the *Cngb1$^{-/-}$* dog retina it shows the presence of truncated CNGB1 in the rod inner segments with none being detectable in the outer segments.

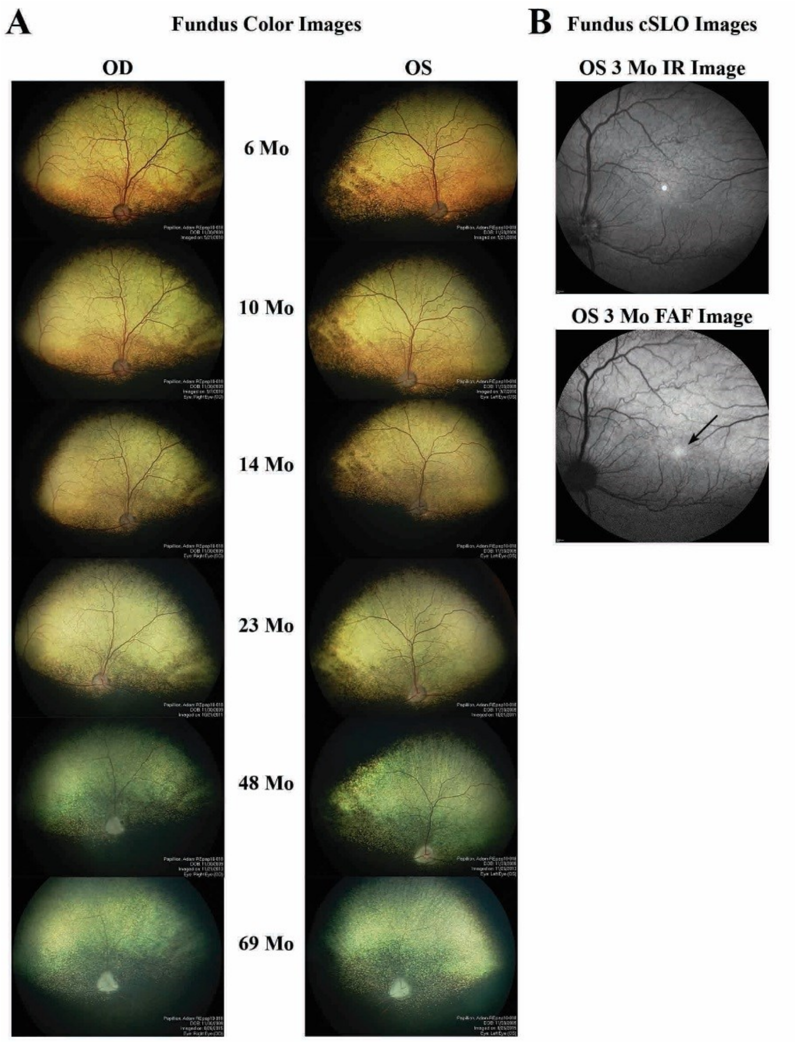

Supplemental Figure 6. (**A**) Color fundus imaging of both eyes of a *Cngb1*$^{-/-}$ dog showing changes in appearance with progression. Initial changes are mild blood vessel attenuation and a generalized tapetal hyporeflectivity. With progression more advanced blood vessel attenuation develops and tapetal hyperreflectivity becomes more apparent. (**B**) cSLO fundus images. The upper infrared (IR) image appears normal but the lower autofluorescent image (488 nm FAF) shows development of an autofluorescent region in the center of the *area centralis* (arrow).

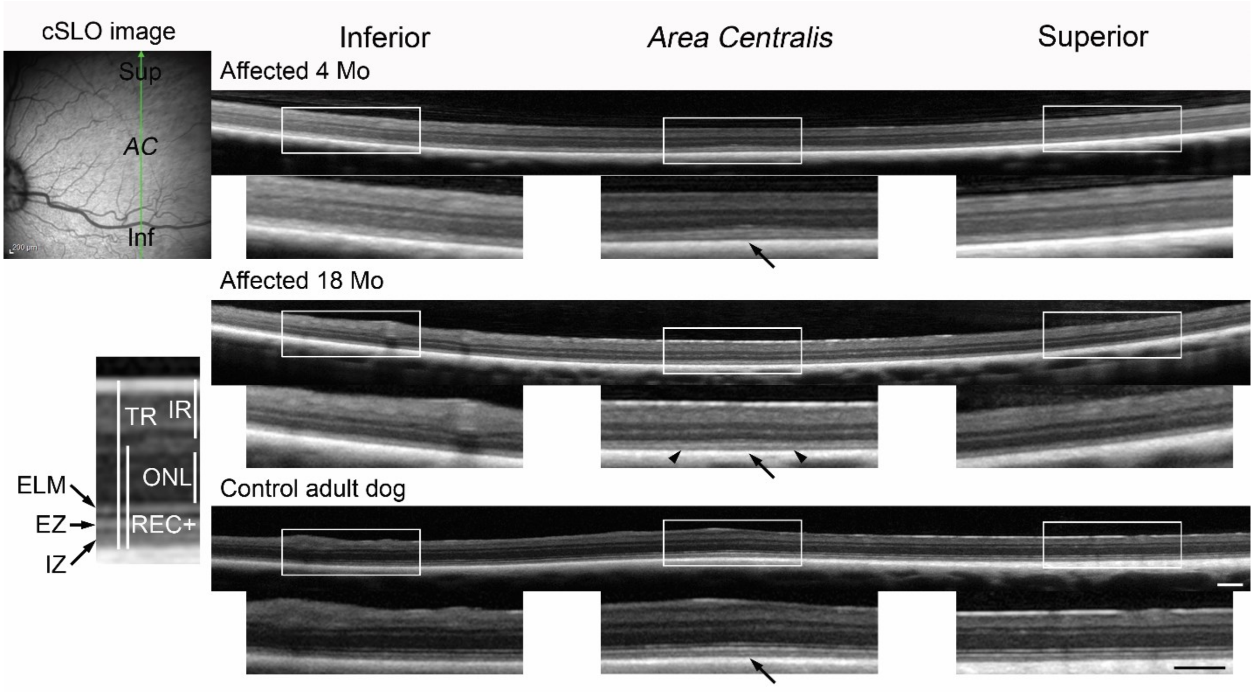

Supplemental Figure 7. *In vivo* cross sectional retinal imaging of 4 month and 18 month old *Cngb1*$^{-/-}$ dogs compared to a normal adult control dog. The cSLO image shows the section imaged for SD-OCT. The regions in the white boxes are shown magnified below each box. Note the loss of definition of zones from the external limiting membrane (ELM) to the interdigitation zone (IZ) in the *Cngb1*$^{-/-}$ retinas starting at the regions at the periphery of the scan. These zones encompass the inner and outer segments of the photoreceptors. The arrows indicate the *area centralis* and the arrowheads indicate a region where there is conserved integrity of the ellipsoid zone (EZ) in the *area centralis*. TR, total retina; IR, inner retina; ONL, outer nuclear layer; REC+, receptor plus; ELM, external limiting membrane; EZ, ellipsoid zone; IZ, interdigitation zone. Size bars = 200μm.

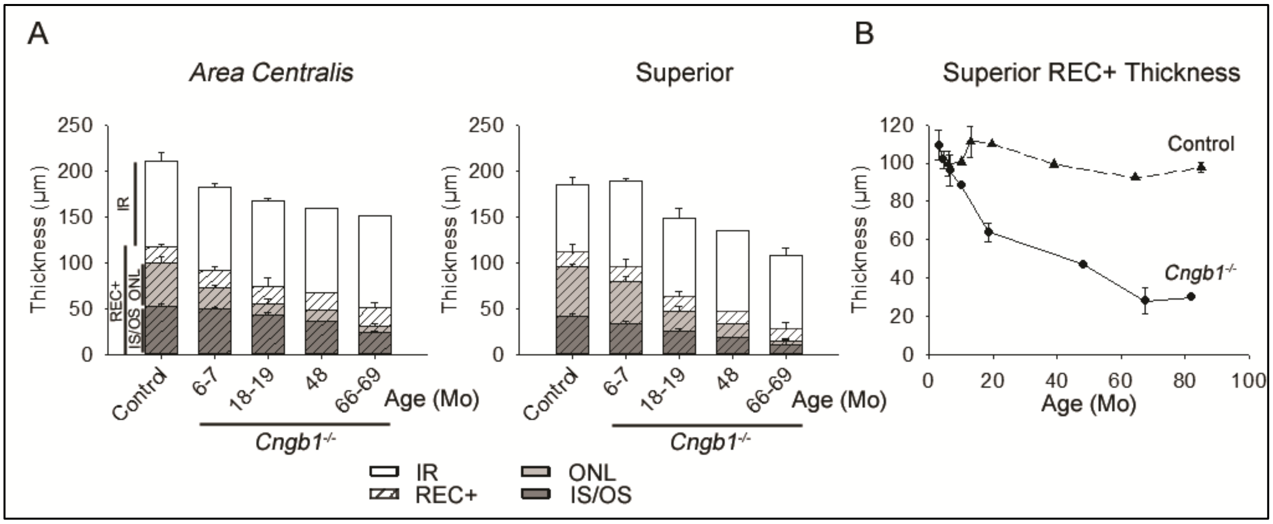

Supplemental Figure 8. (**A**) Bar graphs showing the mean (+/-SD) of SD-OCT measurements of retinal layers in control dogs and different age groups of *Cngb1*$^{-/-}$ dogs in the *area centralis* and a central retinal region superior to the optic nerve head. Note that in the *area centralis* there is slower loss of the IS/OS thickness compared to the dorsal retinal region but initially a relatively faster loss of the ONL (outer nuclear layer). IR, inner retina; REC+, receptor plus; ONL, outer nuclear layer; IS/OS, inner segment/outer segment. Control dogs (n=3), *Cngb1*$^{-/-}$ affected dogs: 6-7 months (n=3), 18-19 months (n=3), 48 months (n=1), 66-69 months (n=2). (**B**) The mean thickness of the REC+ layer from the central superior retina with age in control dogs and *Cngb1*$^{-/-}$ dogs (this can be compared with the graph in Figure 3 for the *Cngb1-X26* mouse). Control dogs: 6 months n=4; 10 months n=1; 13 month n=1; 19.5 month n=1; 39 month n=1; 64.5 months n=1; 85 months n=2. *Cngb1*$^{-/-}$ dogs: 3 month n=2; 4.5 & 6.5 month n=3; 10 month n=1; 18.5 month n=3; 48 month n=1; 67.5 month n=2; 82 month n=1.

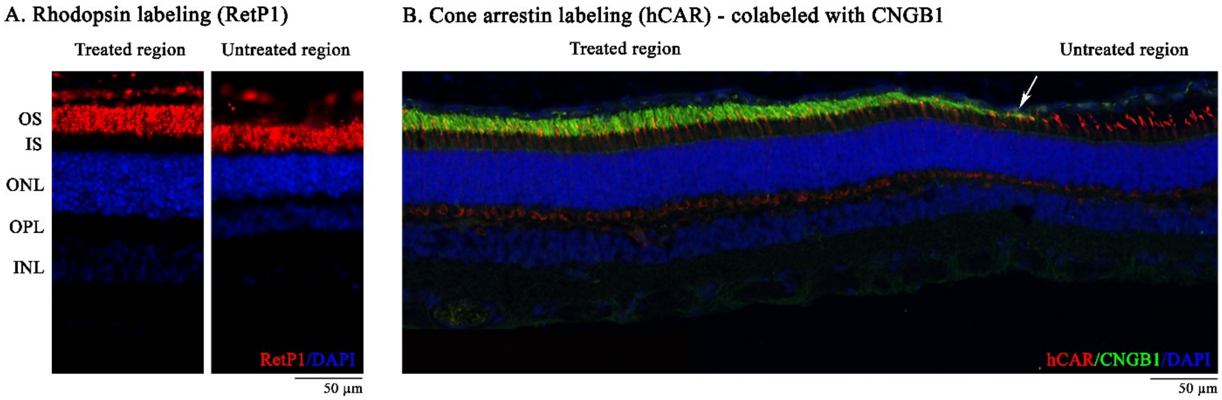

Supplemental Figure 9. IHC sections from *Cngb1*$^{-/-}$ dog (14-055) 23 month post-treatment. **(A)** Rhodopsin is expressed in the rod outer segments of the treated retinal regions and in the untreated region from the same eye it is mislocalized to the rod inner segments. **(B)** C1 is correctly targeted to the outer segments in the treated regions but is not detectable in the untreated region (white arrow indicates the edge of the subretinal injection bleb). hCAR (red - targets all cones) shows presence of normal appearing cones in the treated retina indicating *Cngb1* expression did not have a deleterious effect on cones.

Key: OS, outer segments; IS, inner segments; ONL, outer nuclear layer; OPL, outer plexiform layer; INL, inner nuclear layer.

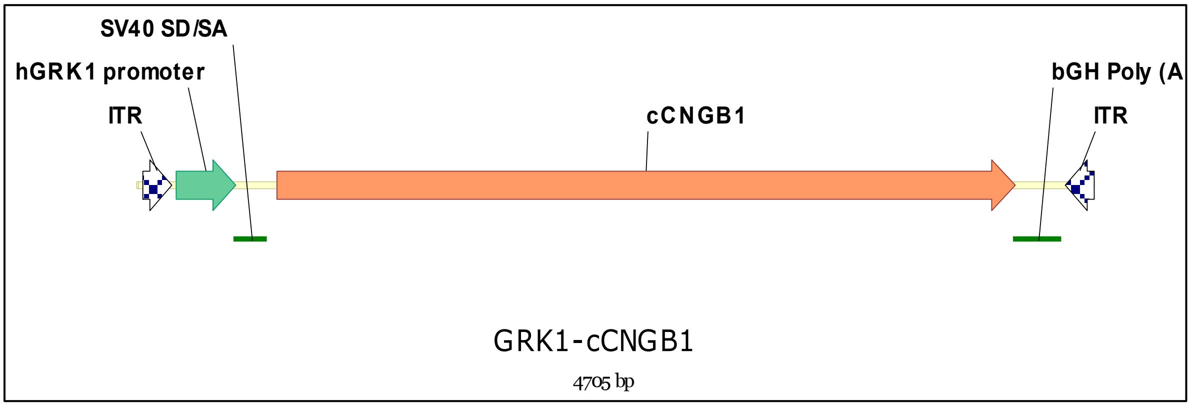

Supplemental Figure 10. Details of the expression cassette for the AAV vector. Feature Map

ITR	Start: 22	End: 164
hGRK1 promoter	Start: 187	End: 478
SV40 SD/SA	Start: 479	End: 642
cCNGB1	Start: 685	End: 4314
bGH Poly (A)	Start: 4316	End: 4551
ITR	Start: 4563	End: 4705

Supplemental Methods.

Minigene splice assay

Minigenes were designed using the *CNGB1* genomic sequence with the accession number NM_001297. The WT and c.1122-9G>A minigenes (1649 bp each) were synthesized by BioCat (Heidelberg, Germany) and were delivered in the pcDNA3.1 (+) standard vector. For RT-PCR analysis, HEK293 cells (from the Mouse Cell Line Authentication Consortium (ATCC):

293 [HEK-293] (ATCC® CRL-1573^{TM}) were transiently transfected using the $CaPO_3$ method. 24 h post transfection, cells were harvested and the RNA was isolated using the RNeasy Mini Kit (QIAGEN) according to the manufacturer's instructions. Subsequent cDNA synthesis was conducted with equal amounts of RNA (200 ng each) using the RevertAid First Strand cDNA Synthesis Kit (Thermo Scientific). PCR was performed using the Herculase II Fusion DNA Polymerase (Agilent Genomics) with following primers:

f1 5'- ccaactcagAGAGCTGTCCCG -3'

f2 5'- GGATTGAAGAGGAGAAAGAAG -3'

r1 5'- CCACACCTACCTCCTGAACTG -3'

PCR conditions were as follows: initial denaturation at 95°C for 1 min; 30 cycles (95°C 20 sec, 60°C 20 sec, 72°C 30 sec) and a final elongation at 72°C for 5 min. The WT and the c.1122-9G>A PCR splice products were extracted, purified, and sequenced. Sequencing was conducted by Eurofins Genomics using the r1 primer.

Supplemental Table 1. Published *CNGB1* mutations and those identified in this study

Previously Published CNGB1 mutations			
Missense	*Nonsense*	*Frameshift*	*Intronic/Splice*
c.1589C> G; p.Pro530Arg (1)	c.262C>T; p.Gln88* (2)	c.2544dupG; p.Leu849Alafs*3 (2)	c.412+1G>A** (3)
c.2284C>T p.Arg762Cys (3, 4)	c.939G>A. p.Trp313*7 (5)	c.2888_2889del; p.Phe963Serfs4* (6)	c.413-1G>A (7)
c.2293C>T. p.Arg765Cys (2, 8, 9)	c.952C>T; p.Gln318* (2)	c.3142-3143insGTGG; p.Ala1048fs13* (2)	c.761+2T>A (2)
c.2957A>T. p.Asn986Ile (2)	c.1896C>A, p.Cys632* (10)	c.3150delG, p.Phe1051Leufs*12 (2, 10)	c.2493-2A>G (11)
c.2978G>T, p.Gly993Val (12)	c.2185C>T; p.Arg729* (2)		c.2493-2_2495delinsGGC (13)
	c.2361C>A. p.Tyr787* (6)		c.3462+1G>A (14)
**Mutations reported in this publication**			
c.2284C>T p.Arg762Cys (rs760373259. MAF 2.50E-5)	c.1896C>A, p.Cys632* (rs774264204. MAF 8.28E-6)	c.522_523insC, p.Lys175Glnfs*4$	c.1122-9G>A$
	c.2508C>A, p.Tyr836*$	c.2544_2545insC, p.Leu849Profs*3$	c.2218-2A>G$
		c.3150delG; p.Phe1051Leufs*12 (rs753353134. MAF 2.49E-5)	

Key: $= First described in this publication. For the mutations identified in this study that had previously been identified the dbSNP, rs number and the minor allele frequency from the Exome Aggregation Consortium database are shown (EXAC: http://exac.broadinstitute.org/). **. Note in the initial publication (Azam et al 2011) (3) this was listed as 412-1G>A – this is incorrect as 412 is the 3' nucleotide of exon 6, therefore it could be either c.412+1G>A (5) or could also be c.413-1G>A which makes it the same as that reported by Saqib et al 2015 (7).

References for previously published CNGB1 mutations

1. Fu Q, Wang F, Wang H, Xu F, Zaneveld JE, Ren H, Keser V, Lopez I, Tuan HF, Salvo JS, et al. Next-generation sequencing-based molecular diagnosis of a Chinese patient cohort with autosomal recessive retinitis pigmentosa. *Invest Ophthalmol Vis Sci.* 2013;54(6):4158-66.
2. Hull S, Attanasio M, Arno G, Carss K, Robson AG, Thompson DA, Plagnol V, Michaelides M, Holder GE, Henderson RH, et al. Clinical Characterization of CNGB1-Related Autosomal Recessive Retinitis Pigmentosa [published online ahead of print January 5, 2017] . *JAMA Ophthalmol.* doi: 10.1001/jamaophthalmol.2016.5213.
3. Azam M, Collin RW, Malik A, Khan MI, Shah ST, Shah AA, Hussain A, Sadeque A, Arimadyo K, Ajmal M, et al. Identification of novel mutations in Pakistani families with autosomal recessive retinitis pigmentosa. *Arch Ophthalmol.* 2011;129(10):1377-8.
4. Bocquet B, Marzouka NA, Hebrard M, Manes G, Senechal A, Meunier I, and Hamel CP. Homozygosity mapping in autosomal recessive retinitis pigmentosa families detects novel mutations. *Mol Vis.* 2013;19:2487-500.
5. Fradin M, Colin E, Hannouche-Bared D, Audo I, Sahel JA, Biskup S, Carre W, Ziegler A, Wilhelm C, Guichet A, et al. Run of homozygosity analysis reveals a novel nonsense variant of the CNGB1 gene involved in retinitis pigmentosa 45. *Ophthalmic Genet.* 2016;37(3):357-9.
6. Xu Y, Guan L, Shen T, Zhang J, Xiao X, Jiang H, Li S, Yang J, Jia X, Yin Y, et al. Mutations of 60 known causative genes in 157 families with retinitis pigmentosa based on exome sequencing. *Hum Genet.* 2014;133(10):1255-71.
7. Saqib MA, Nikopoulos K, Ullah E, Sher Khan F, Iqbal J, Bibi R, Jarral A, Sajid S, Nishiguchi KM, Venturini G, et al. Homozygosity mapping reveals novel and known mutations in Pakistani families with inherited retinal dystrophies. *Sci Rep.* 2015;5:9965.
8. Simpson DA, Clark GR, Alexander S, Silvestri G, and Willoughby CE. Molecular diagnosis for heterogeneous genetic diseases with targeted high-throughput DNA sequencing applied to retinitis pigmentosa. *J Med Genet.* 2011;48(3):145-51.
9. Habibi I, Chebil A, Falfoul Y, Allaman-Pillet N, Kort F, Schorderet DF, and El Matri L. Identifying mutations in Tunisian families with retinal dystrophy. *Sci Rep.* 2016;6:37455.
10. Nishiguchi KM, Tearle RG, Liu YP, Oh EC, Miyake N, Benaglio P, Harper S, Koskiniemi-Kuendig H, Venturini G, Sharon D, et al. Whole genome sequencing in patients with retinitis pigmentosa reveals pathogenic DNA structural changes and NEK2 as a new disease gene. *Proc Natl Acad Sci U S A.* 2013;110(40):16139-44.
11. Maria M, Ajmal M, Azam M, Waheed NK, Siddiqui SN, Mustafa B, Ayub H, Ali L, Ahmad S, Micheal S, et al. Homozygosity mapping and targeted sanger sequencing reveal genetic defects underlying inherited retinal disease in families from pakistan. *PLoS One.* 2015;10(3):e0119806.
12. Schorderet DF, Iouranova A, Favez T, Tiab L, and Escher P. IROme, a new high-throughput molecular tool for the diagnosis of inherited retinal dystrophies. *BioMed Res Int.* 2013;2013:198089.
13. Maranhao B, Biswas P, Gottsch AD, Navani M, Naeem MA, Suk J, Chu J, Khan SN, Poleman R, Akram J, et al. Investigating the Molecular Basis of Retinal Degeneration in a Familial Cohort of Pakistani Decent by Exome Sequencing. *PLoS One.* 2015;10(9):e0136561.
14. Kondo H, Qin M, Mizota A, Kondo M, Hayashi H, Hayashi K, Oshima K, Tahira T, and Hayashi K. A homozygosity-based search for mutations in patients with autosomal recessive retinitis pigmentosa, using microsatellite markers. *Invest Ophthalmol Vis Sci.* 2004;45(12):4433-9.

Supplemental Table 2 Gene therapy in *Cngb1*$^{-/-}$ dogs

Dog	Eye	Vector	Dose per eye (volume)	Age at Injection (weeks)	Duration following injection
14-056	OD	AAV5-GRK1-cCNGB1	1.5×10^{12} (300µl)	22	3 months
14-056	OS	AAV5-GRK1-cCNGB1	1.5×10^{12} (300µl)	22	3 months
15-046	OD	AAV5-GRK1-cCNGB1	1.25×10^{12} (250µl)	14.5	3 months
14-097	OD	AAV5-GRK1-cCNGB1	1.5×10^{12} (300µl)	16	6 months
14-033	OD	AAV5-GRK1-cCNGB1	1.75×10^{11} (175µl)	15	14 months
14-033	OS	AAV5-GRK1-cCNGB1	1.0×10^{12} (200µl)	28	9 months
14-055	OD	AAV5-GRK1-cCNGB1	1.5×10^{12} (300µl)	22	23 months
14-055	OS	AAV5-GRK1-cCNGB1	1.5×10^{12} (300µl)	22	23 months
